# Net effects of sodium-glucose co-transporter-2 inhibition in different patient groups: a meta-analysis of large placebo-controlled randomized trials

**DOI:** 10.1016/j.eclinm.2021.101163

**Published:** 2021-10-26

**Authors:** Natalie Staplin, Alistair J. Roddick, Jonathan Emberson, Christina Reith, Alex Riding, Alexa Wonnacott, Apexa Kuverji, Sunil Bhandari, Colin Baigent, Richard Haynes, William G Herrington

**Affiliations:** a Medical Research Council Population Health Research Unit at the University of Oxford, Clinical Trial Service Unit and Epidemiological Studies Unit, Nuffield Department of Population Health (NDPH), University of Oxford, Oxford, UK; b Big Data Institute, Li Ka Shing Centre for Health Information and Discovery, University of Oxford, Oxford, UK; c Cambridge University Hospitals NHS Foundation Trust, Cambridge, UK; d Wales Kidney Research Unit, Cardiff University, Cardiff, UK; e John Walls Renal Unit, Leicester General Hospital, University Hospitals of Leicester, Leicester, UK; f Hull University Teaching Hospitals NHS Trust and Hull York Medical School, Hull, UK; h Oxford Kidney Unit, Churchill Hospital, Oxford, UK; h Health Data Research UK, University of Oxford, Oxford, UK

**Keywords:** Sodium-glucose co-transporter 2 inhibitors, Safety, Heart failure, CKD, Randomized trials

## Abstract

**Background:**

The net absolute effects of sodium-glucose co-transporter-2 (SGLT-2) inhibitors across different patient groups have not been quantified.

**Methods:**

We performed a meta-analysis of published large (>500 participants/arm) placebo-controlled SGLT-2 inhibitor trials after systematically searching MEDLINE and Embase databases from inception to 28th August 2021 (PROSPERO 2021 CRD42021240468).

**Findings:**

Four heart failure trials (n=15,684 participants), four trials in type 2 diabetes mellitus at high atherosclerotic cardiovascular risk (n=42,568), and three trials in chronic kidney disease (n=19,289) were included. Relative risks (RRs) for all cardiovascular, renal and safety outcomes were broadly similar across these three patient groups, and between people with or without diabetes. Overall, compared to placebo, allocation to SGLT-2 inhibition reduced risk of hospitalization for heart failure or cardiovascular death by 23% (RR=0.77, 95%CI 0.73-0.80; n=6658), cardiovascular death by 14% (0.86, 0.81-0.92; n=3962), major adverse cardiovascular events by 11% (0.89, 0.84-0.94; n=5703), kidney disease progression by 36% (0.64, 0.59-0.70; n=2275), acute kidney injury by 30% (0.70, 0.62-0.79; n=1013 events) and severe hypoglycaemia by 13% (0.87, 0.79-0.97; n=1484). There was no effect of SGLT-2 inhibition on risk of non-cardiovascular death (0.93, 0.86-1.01; n=2226), but a net 12% reduction in all-cause mortality remained evident (0.88, 0.84-0.93; n=6188). However, the risk of ketoacidosis was 2-times higher among those allocated SGLT-2 inhibitors compared to placebo (2.03, 1.41-2.93; n=159; absolute excess in people with diabetes ∼0.3/1000 patient years). A small increased risk of urinary tract infection was evident (1.07, 1.02-1.13; n=5384) alongside a known increased risk of mycotic genital infections. Overall, risk of lower limb amputations was increased by 16% (1.16, 1.02-1.31; n=1074), but this risk was largely driven by a single outlying trial (CANVAS).

**Interpretations:**

The relative effects of SGLT-2 inhibition on key safety and efficacy outcomes are consistent across the different studied groups of patient. Consequently, absolute benefits and harms are determined by the absolute baseline risk of particular outcomes, with absolute benefits on mortality and on non-fatal serious cardiac/renal outcomes substantially exceeding the risks of amputation and ketoacidosis in the main patient groups studied to date.

**Funding:**

MRC-UK & KRUK.

**Research in context**

**Evidence before this study**

The first large trials to test the safety of sodium-glucose co-transporter-2 (SGLT-2) inhibitors were conducted among people with type 2 diabetes mellitus (DM) with, or at high risk of, atherosclerotic cardiovascular disease (ASCVD). These trials identified the potential for SGLT-2 inhibitors to reduce cardiovascular risk (particularly heart failure [HF]) and kidney disease progression, but also to increase the risk of ketoacidosis and perhaps lower limb amputation. Large were also initiated in people with established HF or chronic kidney disease (CKD), with or without DM. Reduced efficacy on cardiac and renal outcomes in such patient groups may have been expected. Nevertheless, such trials reported that SGLT-2 inhibitors reduce the risk of cardiac and renal outcomes irrespective of DM status or level of kidney function, and provided reassuring safety data. To obtain precise estimates of clinical safety and assess net absolute benefits across the different studied patient groups requires aggregated results from all these large trials, we performed a systematic review and meta-analysis.

**Added value of this study**

Using data from eleven placebo-controlled clinical trials of people with HF, type 2 DM at high ASCVD risk, or CKD, we found that the relative benefits of SGLT-2 inhibitors on cardiac and renal outcomes were remarkably consistent across these different patient groups, including among people without DM. Overall, risk of cardiovascular death or hospitalization for HF, and risk of kidney disease progression were each reduced by about one-quarter (once trial definitions were standardized). Additionally, allocation to an SGLT-2 inhibitor reduced the risk of acute kidney injury, and severe hypoglycaemia, with no clear effect on risk of bone fracture.

**Implications of all the available evidence**

Placebo-controlled trials of SGLT-2 inhibitors demonstrate their relative effects on efficacy outcomes are remarkably consistent across the different groups of studied people with type 2 DM, HF and CKD. The available trials also show overwhelming evidence for net absolute benefit of SGLT-2 inhibitors in these studied patient groups, and particularly among people with HF and CKD.

Alt-text: Unlabelled box

## 1 Introduction

Sodium-glucose co-transporter-2 (SGLT-2) inhibitors were developed for their effects on blood glucose, and large-scale trials mandated by the US FDA were initiated to assess their cardiovascular safety in populations with type 2 diabetes mellitus (DM) at high atherosclerotic cardiovascular (ASCVD) risk [1]. These trials not only demonstrated that SGLT-2 inhibitors were non-inferior to placebo with respect to cardiovascular safety [2], [3], [4], [5], but some also demonstrated superiority. These results shifted focus to their potential to modify disease risk as compared to solely improving glycaemic control [6]. Subsequent trials in people with documented heart failure (HF) [7], [8], [9], [10] and chronic kidney disease (CKD) [11], [12], [13] have confirmed their efficacy at reducing risk of hospitalization for HF or cardiovascular death, irrespective of the presence of type 2 DM, and an ability to slow CKD progression. SGLT-2 inhibition substantially reduces end-stage kidney disease risk among people with albuminuric diabetic nephropathy [13, 14], and subgroup analyses from one trial suggest there are benefits in certain types of albuminuric non-diabetic causes of CKD [11]. Consequently, SGLT-2 inhibitors are prescribed increasingly among people with HF and CKD.

Adverse effects of SGLT-2 inhibitors have been identified from randomized trials and, in some cases, from post-marketing surveillance. Summaries of product characteristics include warnings about risk of ketoacidosis, lower limb amputations, bone fractures, urinary tract infections and Fournier's gangrene. The relative and/or absolute benefits/hazards of SGLT2-inhibitors on particular outcomes may differ by patient population (e.g. in people with HF versus CKD). This is because different groups of patient may respond differently and/or be at different baseline risk of outcomes. For example, other things being equal, SGLT-2 inhibitors induce less glycosuria in people with CKD [15] than in those without, and less in people without DM [16] than in people with DM.

We aimed to provide reliable patient-specific estimates of the benefits and harms of SGLT-2 inhibitors to help inform clinicians and patients. We therefore planned a meta-analysis of the large placebo-controlled trials aiming to estimate both the relative and absolute effects of SGLT-2 inhibitors for all the key efficacy and safety outcomes, including exploring effects on non-cardiovascular mortality and the impact of different definitions of kidney disease progression. Results are presented overall and separately for the three main different types of patients studied (i.e. people with HF, type 2 DM at high ASCVD risk, and CKD). We also estimate effects in people according to whether they had DM (or not) at trial entry.

## 2 Methods

### 2.1 Literature search and data extraction

An outline protocol was registered in the International Prospective Register of Systematic Reviews (PROSPERO 2021 CRD42021240468) on 4^th^ March 2021, and the Preferred Reporting Items for Systematic Reviews and Meta-Analysis (PRISMA) statement was followed. A systematic search of MEDLINE and Embase databases via OVID was performed to cover the period of inception to 28^th^ August 2021. Titles and abstracts were initially screened, with subsequent screening of full texts and risk of bias assessments (using the Cochrane Risk of Bias 2 tool) were completed independently and in duplicate (see Supplemental Methods). Eligibility required trials to be placebo-controlled, performed in adults, and be large (i.e. to include ≥1000 participants/randomizing ≥500 participants in each arm, thereby minimizing any potential for publication bias to distort findings).

For each included trial, data were extracted after reviewing all the principal [[2], [3], [4], [5], [7], [8], [9], [10], [11], [12], [13], 17] and relevant subsidiary peer-reviewed publications [14, [18], [19], [20], [21], [22], [23], [24], [25], [26]]. The main outcomes were: hospitalization for HF or cardiovascular death; major adverse cardiovascular events (i.e. MACE, cardiovascular death, non-fatal myocardial infarction [MI] or stroke); and kidney disease progression (based on published definitions of categorical outcomes). Assessments of composite outcomes were, wherever possible, supplemented by analyses of each of their constituent components. Death from any cause was also extracted. Information on non-cardiovascular death was also extracted or, where unreported, inferred using information on all-cause and cardiovascular deaths (i.e. included any death not considered to be cardiovascular). The key outcomes used to assess any potential harms of SGLT-2 inhibitors were: acute kidney injury (AKI), ketoacidosis, severe hypoglycaemia, lower limb amputation, bone fracture, urinary tract infection, mycotic genital infections, and Fournier's gangrene. All analysed data were extracted from published sources.

### 2.2 Statistical analysis

Where event rates were not reported, these were estimated from the number of events and participants in each arm and the median duration of follow-up in the trial. Where treatment effects were not reported, log relative risks (RRs) and the associated standard errors (SEs) were estimated from the numbers of events and participants in each arm. Table and figure footnotes specify when such approaches were used.

Inverse-variance-weighted averages of log hazard ratios/RRs were then used to estimate the treatment effects in each patient group and overall [27, 28]. This approach has the desirable property that, at the point of randomization, every participant has the same opportunity to contribute the same amount of statistical information to the meta-analysis as every other participant. Standard chi-square tests for heterogeneity were used to assess whether treatment effects differed between: the three patient groups (i.e. HF, type 2 DM and high ASCVD risk, and CKD); between the trials within each of these patient groups; or between people with and without DM.

Predicted absolute benefits and harms of SGLT-2 inhibitors versus placebo per 1000 patient-years of treatment were estimated for each of the three patient groups and by DM status. The HF groups were additionally separated into trial data among patients with stable HF with reduced ejection fraction (HFrEF), stable HF with preserved ejection fraction (HFpEF), and trial data from recently hospitalized for worsening HF (due to the extremely high absolute risks in the latter group). Absolute effects were estimated by applying the overall RRs (all three patient groups combined) to the average patient group-specific event rate in the placebo arms (first event only). SEs for the numbers of events avoided or caused were estimated from the uncertainty in the RRs. Sensitivity analyses in which observed patient group-specific RRs were applied to patient group event rates were also conducted. Another sensitivity analysis considered the potential impact of important differences in definitions of kidney disease progression used among the trials analysed (i.e. different percent declines in eGFR from baseline: see Supplemental Methods for details of the adjustment derived from analyses in CANVAS [29]). All analyses were performed in SAS version 9.4 (SAS Institute, Cary NY, USA) and R v3.6.2.

### 2.3 Role of funding source

The funders had no role in study design, data collection, data analysis, data interpretation, writing of the report, or the decision to submit for publication. All the authors had access to data and decided to submit the manuscript for publication.

## 3 Results

### 3.1 Eligible trial characteristics

**Figure 1:**
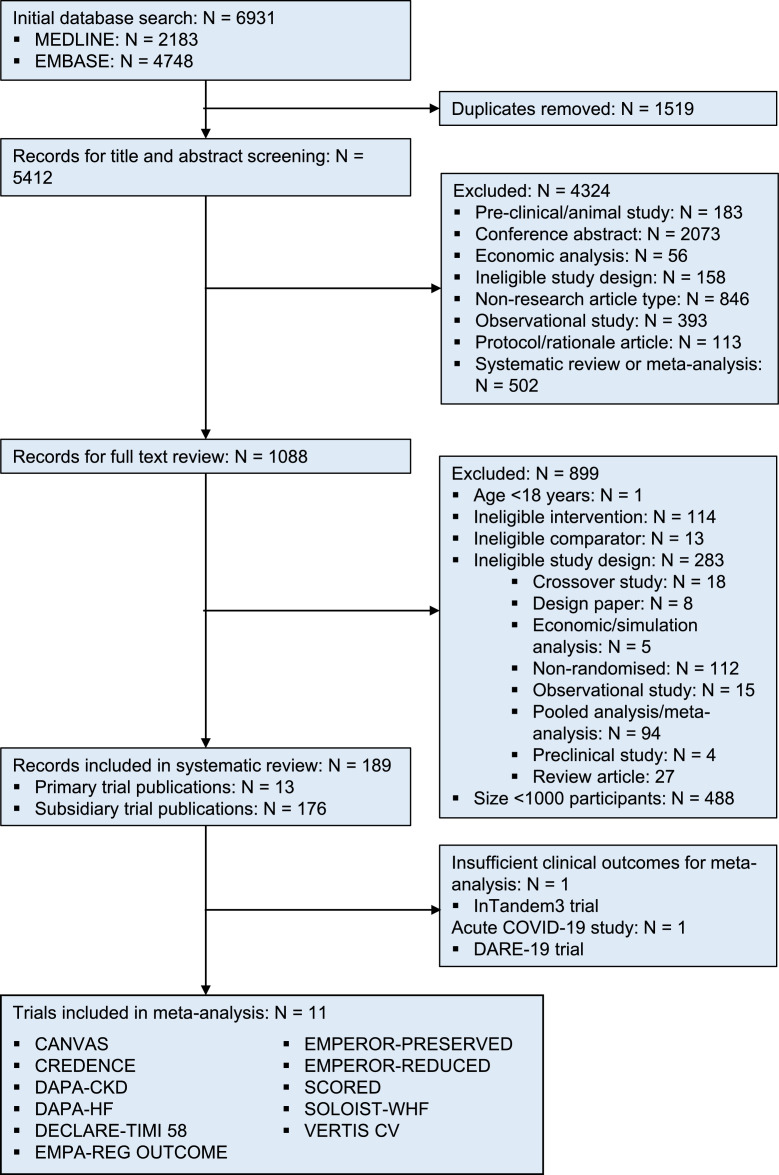
Study selection

6931 potential records were identified, from which 189 publications relating to thirteen large trials met our selection criteria (Figure 1). A trial of 1402 participants with type 1 DM (inTandem3) and a short trial of 1250 people hospitalized with COVID-19 (DARE-19) provided only small numbers of clinical outcomes and so were not included in meta-analyses (Supplemental Methods provide more details/results) [17, 30]. Data for the remaining eleven trials were extracted from their primary publications [[2], [3], [4], [5], [7], [8], [9], [10], [11], [12], [13]] and eleven subsidiary peer-reviewed publications [14, [18], [19], [20], [21], [22], [23], [24], [25], [26], 31]. A total of 77,541 participants were included in meta-analyses: four HF trials randomized 15,684 participants [7], [8], [9], [10], four type 2 DM high-ASCVD risk trials randomized 42,568 participants [2], [3], [4], [5], and three CKD trials randomized 19,289 participants [11], [12], [13]. All trials’ designs were at low risk of bias (Supplemental Table 1).

Table 1 provides the key eligibility criteria, population size, proportion with DM and HF, average estimated glomerular filtration rate (eGFR) and median follow-up for each included trial. Data for people without DM were available from 4479 participants from two HFrEF trials (EMPEROR-REDUCED & DAPA-HF [7, 9]), 3050 from a trial in HFpEF (EMPEROR-PRESERVED [10]), and 1398 from DAPA-CKD [14]. Prior HF was reported in 10-24% of the participants of the type 2 DM high-ASCVD risk trials, and 11-31% of the CKD trials. Average eGFR ranged from 74-85 mL/min/1.73m^2^ in the type 2 DM high-ASCVD risk trials, from 50-66 mL/min/1.73m^2^ in the HF trials, from 43-56 mL/min/1.73m^2^ in the CKD trials. Median follow-up was longest for the type 2 DM high-ASCVD risk trials (range: 3.0-4.2 years), intermediate for the CKD trials (range: 1.3-2.6 years) and shortest for the HF trials (range 0.8-2.2 years).

**Table 1:** Summary of included trials, by patient group

Patient group Trial acronym (drug & daily dose)	Size	Median follow-up, years	Proportion with DM	Proportion with heart failure	Average (SD) eGFR, mL/min/1.73m^2^	Key eligibility criteria
**Heart Failure**						
DAPA-HF (dapagliflozin 10mg)	4744	1.5	42%	100%	Mean: 66 (19)	• Symptomatic chronic HF (class II-IV) with LVEF ≤40% (i.e. reduced ejection fraction) • NT-proBNP ≥600 pg/mL • eGFR ≥30 • Appropriate doses of medical therapy & use of medical devices
EMPEROR-REDUCED (empagliflozin 10mg)	3730	1.3	50%	100%	Mean: 62 (22)	• Class II-IV chronic HF with LVEF ≤40% (i.e. reduced ejection fraction) • NT-proBNP above a certain threshold (stratified by LVEF) • Appropriate doses of medical therapy and use of medical devices
SOLOIST-WHF (sotagliflozin 200-400mg)	1222	0.8	100%	100%	Median: 50	• Hospitalized for HF requiring intravenous therapy (i.e. a HF population with a wide range of LVEFs) • Type 2 DM • eGFR ≥30 • No recent coronary event
EMPEROR-PRESERVED (empagliflozin 10mg)	5988	2.2	49%	100%	Mean: 61 (20)	• Symptomatic chronic HF (class II-IV) with LVEF >40% • Echocardiographic evidence of structural heart disease or hospitalization for heart failure in the last year • NT-proBNP >300 pg/mL (or >900 pg/mL if in AF) • eGFR ≥20 • No recent coronary event

**TYPE 2 DM AT HIGH ASCVD RISK**						
EMPA-REG OUTCOME (empagliflozin 10mg or 25mg)	7020	3.1	100%	10%	Mean: 74 (21)	• Type 2 DM • History of coronary, cerebral or peripheral vascular disease • eGFR ≥30
CANVAS Program (canagliflozin 100-300mg)	10142	2.4	100%	14%	Mean:77 (21)	• Type 2 DM • History of coronary, cerebral or peripheral vascular disease OR age >50y with at least 2 CV risk factors • eGFR ≥30
DECLARE-TIMI 58 (dapagliflozin 10mg)	17160	4.2	100%	10%	Mean: 85 (16)	• Type 2 DM • Age 40y + history of coronary, cerebral or peripheral vascular disease OR age ≥55y in men/≥60y in women with at least 1 CV risk factors • Creatinine clearance ≥60 mL/min
VERTIS CV (ertugliflozin 5 or 15 mg)	8246	3.0	100%	24%	Mean:76 (21)	• Type 2 DM • History of coronary, cerebral or peripheral vascular disease • eGFR ≥30 Type 2 diabetes and

**Chronic kidney disease**						
CREDENCE (canagliflozin 100mg)	4401	2.6	100%	15%	Mean:56 (18)	• Type 2 DM • eGFR 30-90 • uACR 300-5000 mg/g • Stable maximally tolerated RAS blockade
DAPA-CKD (dapagliflozin 10mg)	4304	2.4	68%	11%	Mean:43 (12)	• eGFR 25-75 • uACR 200-5000 mg/g • Stable maximally tolerated RAS blockade, unless documented intolerance
SCORED (sotagliflozin 200-400mg)	10584	1.3	100%	31%	Median: 45	• Type 2 DM • eGFR 25-60 • At least 1 CV risk factor

AF=atrial fibrillation; ASCVD=atherosclerotic cardiovascular disease; CV=cardiovascular; DM=diabetes mellitus; eGFR=estimate glomerular filtration rate (mL/min/1.73m^2^); HF=heart failure; LVEF=left ventricular ejection fraction; NT-proBNP=N-terminal prohormone brain natriuretic peptide; RAS=renin angiotensin system; uACR=urinary albumin:creatinine ratio.

### 3.2 Relative effects of SGLT-2 inhibitors

**Figure 2:**
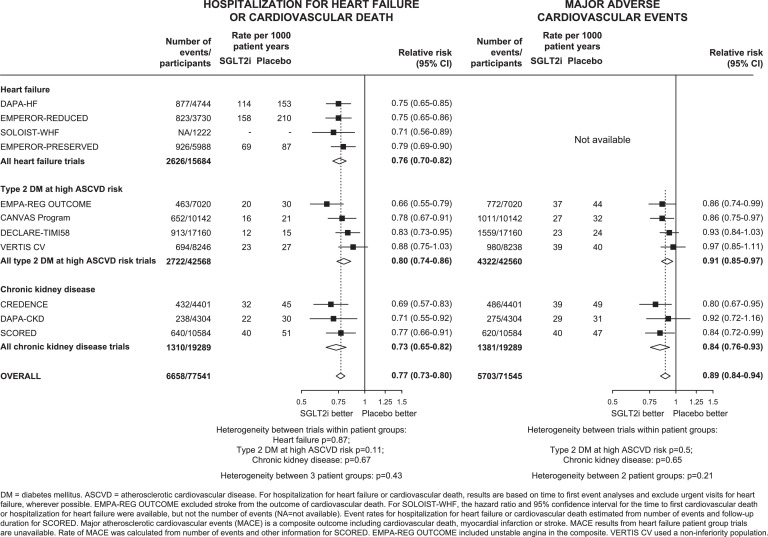
Effects of SGLT-2 inhibitors on (a) HOSPITALIZATION FOR HEART FAILURE OR CARDIOVASCULAR DEATH and (b) MAJOR ADVERSE CARDIOVASCULAR EVENTS, by patient group and by trial

Overall, allocation to SGLT-2 inhibitors compared to placebo reduced the risk of the composite of hospitalization for HF or cardiovascular death by 23% (RR=0.77, 95% CI 0.73-0.80; 6658 events). The relative reductions for the three different patient groups were similar (between population het test p=0.43), with no evidence of heterogeneity between trials within each patient group (all heterogeneity tests p>0.05; Figure 2 & Supplemental Figure 1). Hospitalization for HF was reduced by 32% (RR=0.68, 95% CI 0.64-0.73; 4382 events), and there was no evidence of heterogeneity between patient groups or between trials within each patient group (Supplemental Figure 2).

**Figure 3:**
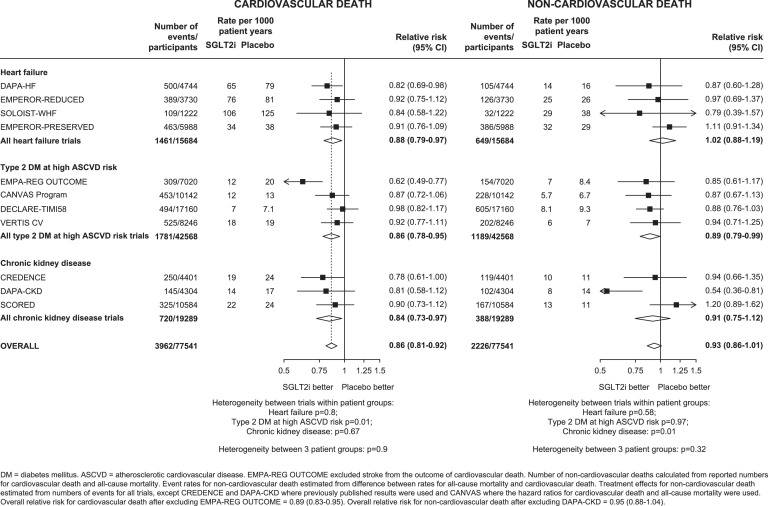
Effects of SGLT-2 inhibitors on (a) CARDIOVASCULAR DEATH and (b) NON-CARDIOVASCULAR DEATH, by patient group and by trial

For the composite of MACE, results from 5703 first such events were available from four trials among patients with type 2 DM at high ASCVD risk and 3 trials among patients with CKD (data from the four HF trials were unavailable). Overall compared to placebo, allocation to SGLT-2 inhibitors reduced risk of MACE by 11% (0.89, 0.84-0.94), with no evidence of heterogeneity of RRs between patient groups or between trials within each patient group (all het tests p>0.05: Figure 2). The relative risk reductions for MACE were driven by a 14% reduction in risk of cardiovascular death (0.86, 0.81-0.92; 3962 events, Figure 3) and an 11% reduction in risk of MI (0.89, 0.82-0.96; 2270 events: Supplemental Figure 3). There was no significant effect on stroke (0.94, 0.85-1.04; 1422 events).

For cardiovascular death, the effects of allocation to an SGLT-2 inhibitor appeared larger in the EMPA-REG OUTCOME trial compared to the other trials in people with type 2 DM at high ASCVD risk (het p=0.01; Figure 3), but there was no heterogeneity of effects among HF trials (het p=0.80) or CKD trials (het p=0.67). There was also no evidence that RRs differed between the three patient groups (het p=0.90).

For non-cardiovascular death, overall there was no significant effect of SGLT-2 inhibition risk compared to placebo (0.93, 0.86-1.01; 2226 events: Figure 3). The significant reduction in risk of non-cardiovascular death in DAPA-CKD appeared heterogeneous to the other CKD trials (het p=0.01). There was no evidence that RRs differed between trials within the other trial populations (het p=0.58 and 0.97 respectively), or between the three patient groups (het p=0.32). SGLT-2 inhibition reduced the risk of death from any cause by 12% (0.88, 0.84-0.93; 6188 events), with similar relative effects observed in each of the patient groups studied (between population het test p=0.65, Supplemental Figure 4).

**Figure 4:**
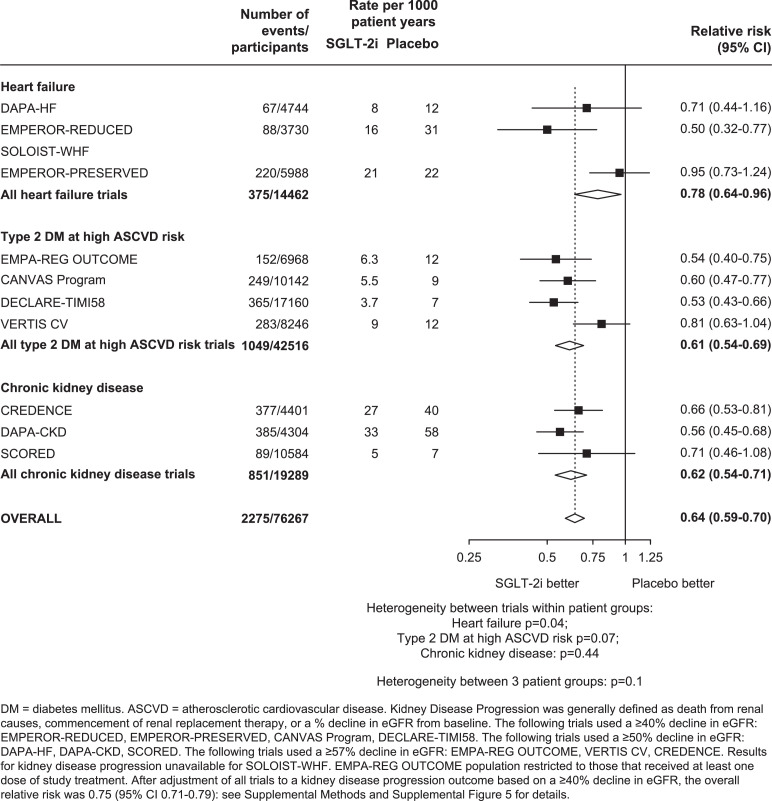
Effects of SGLT-2 inhibitors on KIDNEY DISEASE PROGRESSION, by patient group and by trial

For kidney disease progression, as compared to placebo, allocation to SGLT-2 inhibitors reduced the risk of kidney disease progression by 36% (0.64, 0.59-0.70; 2275 events; Figure 4). In a sensitivity analysis in which trial results were adjusted to reflect estimated effects on the same outcome of a ≥40% decline in eGFR from baseline, the results indicated that there was a 25% reduction (0.75, 0.71-0.79) in risk of kidney disease progression when defined in this way. After applying this adjustment, there was evidence to suggest smaller effects on kidney disease progression in VERTIS CV when compared to other trials conducted in people with type 2 DM at high ASCVD risk (het p=0.0001), but no clear evidence of heterogeneity of effects between the trials conducted in people with HF (het p=0.05) or CKD (het p=0.08; Supplemental Figure 5).

**Figure 5:**
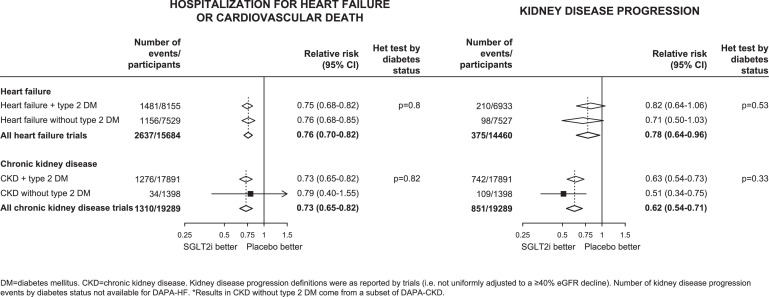
Effects of SGLT-2 inhibitors on (a) HOSPITALIZATION FOR HF OR CARDIOVASCULAR DEATH and (b) KIDNEY DISEASE PROGRESSION, by type 2 diabetes mellitus (DM) status

Three trials in patients with HF (n=7529) and one in patients with CKD (n=1398) have included people without DM at baseline. The effect of allocation to SGLT-2 inhibitors on risk of hospitalization for HF or cardiovascular death appeared similar irrespective of whether DM was present (het tests by DM status p=0.80 for the HF trials & 0.82 for the CKD trials). This was also the case for kidney disease progression as defined by the individual trials (het tests by DM status p=0.53 & 0.33, respectively: Figure 5). These heterogeneity tests by DM status were similar after adjustment of RRs to reflect effects on the harmonised outcome of a ≥40% decline in eGFR (p=0.56 & 0.17, respectively).

**Figure 6:**
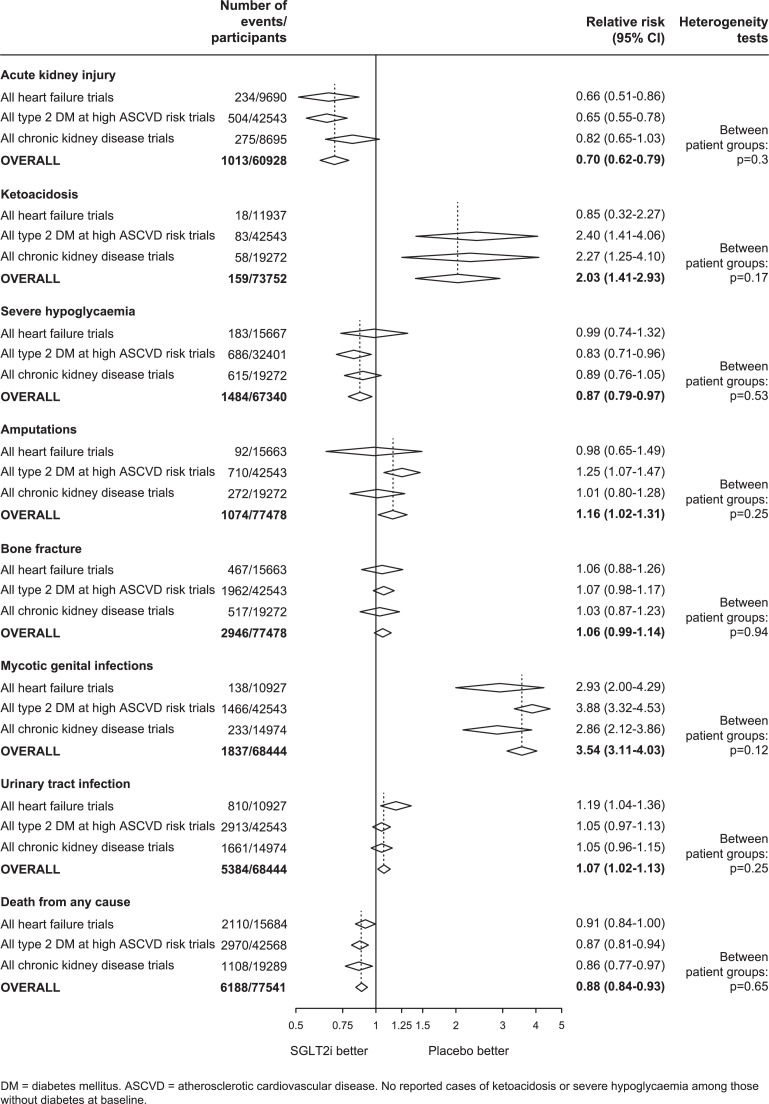
Effect of SGLT-2 inhibitors on SAFETY OUTCOMES, by patient group

Figure 6 provides analyses of the key safety assessments overall and for each patient group considered separately, and Supplemental Figures 6-10 provide corresponding analyses by trial. Allocation to SGLT-2 inhibitors reduced the risk of AKI by 30% compared to placebo (0.70, 0.62-0.79; 1013 events), and there was no evidence the RRs varied between or within trial populations (all het test p>0.05).

Overall, the risk of ketoacidosis was 2-times higher among those allocated SGLT-2 inhibitors compared to placebo (2.03, 1.41-2.93; 159 events), and there was no evidence RRs varied among different patient groups (all het test p>0.05). In the large inTandem3 trial conducted in people with type 1 DM, the relative hazard of ketoacidosis appeared at least as large as the aggregated results from the other trials (sotagliflozin 21 participants [30/1000 patient-years] versus 4 participants allocated placebo [6/1000patient-years]) [17]. Estimates of effects on absolute risk are provided in a section below. Allocation to SGLT-2 inhibitors reduced the risk of severe hypoglycaemia by 13% (0.87, 0.79-0.97; 1484 events), again without heterogeneity of effects in the different patient groups studied (all het test p>0.05: Supplemental Figure 6). No cases of severe hypoglycaemia or ketoacidosis have been reported among participants without DM.

Allocation to SGLT-2 inhibitors increased the risk of lower limb amputation by 16% (1.16, 1.02-1.31; 1074 events). Although there was no evidence that RRs differed between trial patient groups (between population het test p=0.25), the effects on amputation appeared larger in the CANVAS trial than in the other type 2 DM-high ASCVD risk trials (between trial het test p=0.02). The overall RR for amputation attenuated to 6% and was no longer nominally statistically significant after excluding results of CANVAS (1.06, 0.93-1.21: Supplemental Figure 7). For bone fracture, there was no significant effect of SGLT-2 inhibitors compared to placebo overall (1.06, 0.99-1.14; 2946 events), and no evidence for any significant differences between the patient groups studied (all het test p>0.05).

Overall, the risk of mycotic genital infections was 3.54-times higher among those allocated an SGLT-2 inhibitor compared to placebo (3.54, 3.11-4.03; 1837 events), but these infections rarely led to severe complications and there were too few cases of Fournier's gangrene to estimate RRs reliably (Supplemental Figure 9). However, there was only a small 7% increased risk of urinary tract infection, with no evidence that any particular patient group differed in susceptibility to such an outcome (1.07, 1.02-1.13; 5384 events; all het test p>0.05: Supplemental Figure 8).

### 3.3 Estimates of absolute effects of SGLT-2 inhibitors

Table 2 provides estimates of absolute benefits and harms of SGLT-2 inhibitors for the different trial patient groups, including standard errors for these estimates. Risk of hospitalization for HF was particularly high in SOLOIST-WHF (in which patients had recently been hospitalized for worsening HF), so results were considered separately for the different HF populations. For every 1000 patients treated for one year, allocation to an SGLT-2 inhibitor in patients with HFrEF was estimated to prevent 7 first kidney disease progression (unadjusted for differences in definitions) and 6 serious AKI events, 39 HF hospitalizations, and 11 cardiovascular deaths, and cause 0.6 amputations. Compared to HFrEF, the absolute benefits on cardiovascular outcomes in HFpEF were about half the size (19 HF hospitalizations, and 5 cardiovascular deaths prevented per 1000 patient years of treatment with an SGLT-2 inhibitor). For every 1000 patients with recent hospitalization with worsening HF, allocation to an SGLT-2 inhibitor was estimated to prevent 204 HF hospitalizations and 17 cardiovascular deaths in the course of a year.

**Table 2:** Predicted absolute benefits and harms of SGLT-2 inhibitors per 1000 patient-years of treatment, by patient group

	Absolute rates and effects per 1000 patient years									
	STABLE HEART FAILURE				RECENTLY HOSPITALIZED FOR WORSENING HEART FAILURE		TYPE 2 DIABETES MELLITUS AT HIGH ATHEROSCLEROTIC CARDIOVASCULAR RISK		ALBUMINURIC CHRONIC KIDNEY DISEASE	
	REDUCED EJECTION FRACTION		PRESERVED EJECTION FRACTION							
	Event rate	Events avoided/ caused (SE) in SGLT-2i arms	Event rate	Events avoided/ caused (SE) in SGLT-2i arms	Event rate	Events avoided/ caused (SE) in SGLT-2i arms	Event rate	Events avoided/ caused (SE) in SGLT-2i arms	Event rate	Events avoided/ caused (SE) in SGLT-2i arms
**Efficacy Outcomes**										
Hospitalization for heart failure	123	-39 (3)	60	-19 (1)	639	-204 (14)	10	-3 (0.2)	20	-6 (0.4)
Myocardial infarction	-	-	-	-	-	15	-2 (0.5)		9	-1 (0.3)
Cardiovascular death	80	-11 (2)	38	-5 (1)	125	-17 (3)	13	-2 (0.4)	21	-3 (0.6)
Kidney disease progression	20	-7 (0.6)	22	-8 (0.6)	-	-	9	-3 (0.3)	49	-18 (1)
Acute kidney injury	19	-6 (0.9)	-	-	59	-18 (3)	4	-1 (0.2)	15	-5 (0.7)

**Safety Outcomes**										
Ketoacidosis	-	-	-	-	-	-	0.2	0.3 (0.1)	0.3	0.3 (0.1)
Amputation	4	0.6 (0.3)	4	0.5 (0.3)	2	0.3 (0.2)	4	0.7 (0.3)	9	1 (0.7)

Patient group specific absolute effects estimated by applying the overall relative risk to the average event rate in the placebo arms (first event only). For the heart failure patient groups the placebo event rates were estimated separately for trials of stable heart failure with reduced ejection fraction (i.e. EMPEROR-REDUCED & DAPA-HF) versus stable heart failure with preserved ejection fraction (i.e. EMPEROR-PRESERVED) versus recent hospitalization for heart failure (i.e. SOLOIST-WHF). Standard errors (SE) in the numbers of events avoided or caused estimated from uncertainty in the relative risks. Kidney disease progression definitions were as reported by trials (i.e. not uniformly adjusted to a ≥40% eGFR decline). Data on acute kidney injury not available in trials of heart failure with preserved ejection fraction. There were too few ketoacidosis events to estimate absolute effects in heart failure patient groups.

The corresponding absolute benefits/harms for patients with type 2 DM at high ASCVD risk were: 3 first episodes of kidney disease progression and 1 serious AKI event, 3 HF hospitalizations, 2 cardiovascular deaths, and 2 MIs per 1000 patient-years of treatment were avoided at the cost of 0.7 additional amputations and 0.3 ketoacidosis events. For patients with CKD, each 1000 patient-years of treatment with an SGLT-2 inhibitor was estimated to prevent 18 first kidney disease progression and 5 serious AKI events, 6 HF hospitalizations, 3 cardiovascular deaths, and 1 MI, and cause 1 additional amputation and 0.3 ketoacidosis events. Analyses using patient group-specific RRs yielded similar findings (Supplemental Table 2).

In analyses restricted to people without DM, for every 1000 participants treated for one year, allocation to an SGLT-2 inhibitor was estimated to prevent 33 HF hospitalizations or cardiovascular deaths in people with HFrEF, and prevent 15 such outcomes in corresponding analyses for HFpEF (Supplemental Table 3). In albuminuric CKD without DM, 19 first kidney disease progression events and 3 HF hospitalizations or cardiovascular deaths were estimated to be prevented per 1000 patients treated for a year. In people without DM, there were too few ketoacidosis and amputation events to estimate any potential hazard of SGLT-2 inhibitors in this patient group.

## 4 Discussion

Our main aim was to estimate the balance of benefits and hazards of SGLT-2 inhibitors in the different patient groups recruited into placebo-controlled SGLT-2 inhibitor trials to date. We found that, in general, the relative effects of SGLT-2 inhibitors on mortality, key efficacy and most safety outcomes were similar in patients with HF, type 2 DM at risk of high ASCVD, and CKD. The estimated relative effects of SGLT-2 inhibitors in patients with stable HF or with CKD were also similar in size in people with and without DM. In such a situation, the overall relative risk reductions estimated from meta-analysis are likely to be the most reliable (and precise) estimate of relative effects of SGLT-2 inhibitors in a given patient group. These overall aggregated results showed SGLT-2 inhibitors reduced risk of cardiovascular death or hospitalization for HF, and risk of kidney disease progression (defined as a ≥40% decline in eGFR) by about 25%. SGLT-2 inhibitors also reduced the risk of AKI and modestly reduced risk of severe hypoglycaemia, with no clear effect on bone fracture or non-cardiovascular death. SGLT-2 inhibitors are known to increase the risk of mycotic genital infection but serious complications are rare. A marginally increased risk of urinary tract infections is evident, an effect which is only now detectable following the availability of over 5000 such infections in the large trials. Among people with DM, risk of ketoacidosis was increased with a relative risk of 2.0, but uncertainty around this estimate remains due to the limited number of events. The risk of lower limb amputation was increased by about 15-20%, but this risk was largely driven by a single outlying trial (CANVAS). However, despite these uncertainties when quantifying risk of SGLT-2 inhibition, the absolute excess risk of ketoacidosis and amputation was clearly about an order of magnitude smaller than the absolute benefits on cardiac and renal outcomes in people with type 2 DM at high ASCVD risk or with CKD, and the absolute cardiac benefits were nearer two orders of magnitude greater in people with HF.

The absolute risks of the key efficacy and safety outcomes varied substantially across, and also sometimes within, the different studied patient groups. Consequently, there was variation in absolute effects of SGLT-2 inhibitors across patient groups. For example, absolute benefits on HF hospitalization ranged from ∼20 to ∼40 fewer hospitalizations for HF per 1000 patient-years of treatment among those with stable HFpEF and HFrEF, respectively. This increased to about ∼200 fewer such events in those with recent hospitalization for worsening HF, and was as low as ∼3 and ∼6 fewer such hospitalizations per 1000 patient-years of treatment in people with type 2 DM at high ASCVD risk and CKD, respectively.

Patients with CKD were intermediate in their absolute risk of HF hospitalization but were at highest risk of kidney disease progression. They therefore experienced large absolute renal benefits, including ∼20 kidney progression events for every 1000 patients treated for a year. With longer follow-up, these renal benefits may translate into clinically important reductions in the need for dialysis or kidney transplantation. There was also a reduction of ∼5 serious AKI per 1000 patients treated for a year in people with CKD. Reduced risks of AKI risk were evident in patients with HF despite multiple co-prescription of diuretics, renin-angiotensin system blockade and mineralocorticoid receptor antagonists [18]. This is consistent with volume depletion not being a consistent hazard in the large trials. It is noteworthy that diarrhoea, hypotension and volume depletion have been reported in trials testing sotagliflozin, perhaps due to its greater ability to inhibit of gut SGLT-1 compared the more selective SGLT-2 inhibitors tested in the other large trials [8, 12].

Data from nearly 9000 participants without DM from subgroups of three trials in stable HF [7, 9, 10, 26, 31] trials and one CKD trial [14] are consistent with the RRs for key efficacy outcomes being similar to RRs in people with DM, despite lower blood glucose levels. Absolute risks of these efficacy outcomes were, on average, slightly lower in people without DM compared to those with DM within the respective patient groups. However, the lack of any reported severe hypoglycaemia or diabetic ketoacidosis and the exceedingly low number of amputations in people without DM (two reported in EMPEROR-REDUCED [19] and one in DAPA-CKD [14]) meant that benefit:risk ratios are predicted to be exceedingly high among those without DM who have HF or albuminuric CKD (Supplemental Table 3).

In people with type 1 DM, the effects on HbA1c and DM-related events have been assessed in trials, but there are insufficient data to assess effects on cardiovascular and renal clinical outcomes. The 24-week inTandem3 trial highlighted the particularly high absolute excess risk of ketoacidosis in this patient group (a 24/1000 patient-years excess) [17]. Combined results from the EASE trials of empagliflozin yielded similar findings [32], so the absolute benefit:risk ratios are likely to be more finely balanced in people with type 1 DM than in the better-studied patient groups.

This meta-analysis takes into account all the available large-scale randomized evidence from ∼78,000 people recruited into eleven large placebo-controlled clinical trials. Nevertheless there are some limitations. First, meta-analysis is based on summary statistics, so it has not been possible to explore effects on recurrent events, nor to standardize outcome definitions (e.g. we extrapolated estimates from a single trial to adjust kidney disease progression to a ≥40% decline in eGFR from baseline [29]). Second, further data on in HFpEF and certain CKD patient groups are awaited [25, 33] and these ongoing trials will provide more information in people without DM. Third, our absolute effect estimates are specific to the recruited trial populations, where eligibility criteria select for low risk of safety outcomes and high risk of the primary outcome. Relative risks are more generalizable, and so, in routine clinical practice, absolute benefits or harms of SGLT-2 inhibitors could be estimated for an individual by calculating their absolute risk for an event using an established risk score and then applying the overall RRs for the relevant outcome from the presented meta-analyses.

In conclusion, large placebo-controlled trials of SGLT-2 inhibitors have demonstrated that the relative effects of SGLT-2 inhibitors on mortality and on other key efficacy outcomes are remarkably consistent across the different studied patient groups, and similar in people with and without DM. Absolute benefits and harms are therefore determined by the absolute risks of particular outcomes. In the large trial populations studied to date, the absolute excess risks of amputation and ketoacidosis with SGLT-2 inhibitors are approximately an order of magnitude lower than the absolute benefits on cardiac and renal outcomes in people with type 2 DM at high ASCVD risk, or with CKD, and approaching two orders of magnitude smaller for people with recently hospitalization with HF. The low risk of amputation and of ketoacidosis in people without DM suggests that the benefit-to-risk ratios may be particularly favourable in those at risk of HF complications or of CKD progression despite the absence of DM.

## 5 CONTRIBUTORS

WGH conceived the study and developed its design with NS, AJR, CB & RH. AJR performed the systematic literature search with AR, AW, AK, SB & WGH. WGH, RH & AJR extracted data. NS performed statistical analyses and additional checks. WGH, AJR & NS wrote the first draft of the manuscript with all authors contributing to data interpretation and revision of the manuscript.

## Declaration of Competing Interest

CTSU has a staff policy of not accepting honoraria or other payments from the pharmaceutical industry, expect for the reimbursement of costs to participate in scientific meetings. NS, JE, CR, CB, RH, and WGH report a grant paid to their institution by Boehringer Ingelheim. SB reports honoraria from Astra Zeneca and Napp. CR is on the Board of Directors for CDISC and reports funding from the British Heart Foundation. RH and WGH also report grants paid to their institution from Novartis, Roche, and Regeneron. CB, CR, and WGH report funding from MRC-UK. WGH reports personal funding from Kidney Research UK and is co-chair of the UK Kidney Association's clinical guideline for use of SGLT-2 inhibitors in adults with chronic kidney disease. All the other authors report no conflicts.

## Appendix Supplementary materials

Image, application 1
