# Supplementary material for: Net effects of sodium-glucose co-transporter-2 inhibition in different patient groups: a meta-analysis of large placebo-controlled randomized trials
Source: eClinicalMedicine. 2021 Oct 26;41:101163. doi: 10.1016/j.eclinm.2021.101163 (PMC8571171; doi:10.1016/j.eclinm.2021.101163)
Supplement: Supplementary file 1 [file mmc1.pdf]

Supplemental Online Content

NET EFFECTS OF SODIUM-GLUCOSE CO-TRANSPORTER-2 INHIBITION IN DIFFERENT PATIENT GROUPS: A META-ANALYSIS OF LARGE PLACEBO-CONTROLLED RANDOMIZED TRIALS

Supplemental Methods: Supplemental methods

Supplemental TABLE 1: Quality Assessment

Supplemental TABLE 2 Predicted absolute benefits and harms of SGLT-2 inhibitors per 1000 patient-years of treatment, by patient group (sensitivity analysis using patient group-specific RRs)

Supplemental TABLE 3: Predicted absolute benefits and harms of SGLT-2 inhibitors per 1000 patient years of treatment in A) STABLE HEART FAILURE and B) ALBUMINURIC CHRONIC KIDNEY DISEASE, by diabetes status

Supplemental FIGURE 1: Effects of SGLT -2 inhibitors on (a) HOSPITALIZATION FOR HF OR CARDIOVASCULAR DEATH and (b) KIDNEY DISEASE PROGRESSION, by diabetes status and by trial

Supplemental FIGURE 2: Effects of SGLT -2 inhibitors on HOSPITALIZATION FOR HF, by patient group and by trial

Supplemental FIGURE 3: Effects of SGLT -2 inhibitors on (a) MYOCARIDAL INFARCTION and (b) STROKE, by patient group and by trial

Supplemental FIGURE 4: Effects of SGLT-2 inhibitors on ALL-CAUSE MORTALITY, by patient group and by trial

Supplemental FIGURE 5: Effects of SGLT -2 inhibitors on KIDNEY DISEASE PROGRESSION, by patient group and by trial, before and after applying adjustment factor for the different definitions of percent decline in eGFR

Supplemental FIGURE 6: Effects of SGLT -2 inhibitors on (a) KETOACIDOSIS and (b) SEVERE HYPOGLYCAEMIA, by patient group and by trial

Supplemental FIGURE 7: Effects of SGLT -2 inhibitors on (a) LOWER LIMB AMPUTATION and (b) BONE FRACTURE, by patient group and by trial

Supplemental FIGURE 8: Effects of SGLT-2 inhibitors on URINARY TRACT INFECTION, by patient group and by trial

Supplemental FIGURE 9: Effects of SGLT -2 inhibitors on (a) MYCOTIC GENITAL INFECTION and (b) FOURNIER'S GANGRENE, by patient group and by trial

Supplemental FIGURE 10: Effects of SGLT-2 inhibitors on ACUTE KIDNEY INJURY, by patient group and by trial

This supplemental material has been provided by the authors to give readers additional information about their work.

NET EFFECTS OF SODIUM-GLUCOSE CO-TRANSPORTER-2 INHIBITION IN DIFFERENT PATIENT GROUPS: A META-ANALYSIS OF LARGE PLACEBO-CONTROLLED RANDOMIZED TRIALS

SUPPLEMENTAL METHODS

Literature search methods

This systematic review and meta-analysis is reported according to the Preferred Reporting Items for Systematic Reviews and Meta-Analyses (PRISMA) guidance (1), and was registered on PROSPERO before the final database search (PROSPERO ID: CRD42021240468). The systematic database search of MEDLINE and EMBASE databases was undertaken via OVID using a pre-specified search strategy. The search was piloted on 16th February 2021, and the final database search was undertaken on 28th August 2021. The search terms used for interrogating the MEDLINE and EMBASE databases are summarised below. Validated filters for randomised controlled trials in MEDLINE and EMBASE were identified from the Cochrane Handbook for Systematic Reviews of Interventions were used (2).

Identified records were downloaded into a dedicated database and screened for duplicates. An initial screen of titles and abstracts was undertaken by a single reviewer (AJR), with subsequent full text screening undertaken by two reviewers independently and in duplicate (two of AJR, AR, AK, AW, SB), using a piloted spreadsheet. All screening was undertaken against predetermined inclusion criteria. Discrepancies between reviewers were resolved by a third reviewer (WGH). The database included main publications and all identified subsidiary peer-reviewed publications.

The inclusion criteria were as follows:

- Parallel-group randomized controlled trial in adults
- Randomization of at least 1000 participants to an SGLT-2 inhibitor (including SGLT-1/SGLT-2 inhibitors) versus placebo (including at least 500 participants in each group)
- Reporting any of the pre-specified main efficacy outcomes and any of the pre-specified safety outcomes

Where multiple reports from the same study were identified, these were collated by reference to the study acronym, or the National Clinical Trials (NCT) reference number. Reference lists of included studies and contemporary meta-analyses were screened for additional relevant studies.

Outcomes

Pre-specified outcomes comprised the following efficacy and safety outcomes.

Efficacy outcomes:

- Hospitalization for heart failure (HF) or cardiovascular death, overall and by components

- Major adverse cardiovascular events (MACE, defined as cardiovascular death, non-fatal myocardial infarction, or non-fatal stroke), overall and by components
- Kidney disease progression (based on published definitions of categorical outcomes)
- Death from cardiovascular and non-cardiovascular causes considered separately (with effects on non-cardiovascular causes estimated from cardiovascular and all-cause mortality results when non-cardiovascular death results were not reported)

Safety outcomes:

- Acute kidney injury
- Ketoacidosis
- Severe hypoglycaemia (based on individual trial definitions)
- Lower limb amputation
- Bone fracture
- Mycotic genital infections and Fournier's gangrene
- Urinary tract infection

Data extraction

For each trial, relevant results were identified from the main (3-13) and/or subsidiary peer-reviewed publications (14-23) and extracted into spreadsheets for checking. Data extraction included each trial's main eligibility criteria; follow-up duration; selected participant characteristics (proportion with diabetes mellitus (DM), HF and the presented "average" kidney function); number of events and participants included in reported comparisons; event rate per 1000 patient years in each arm; and any reported hazard ratio for the effect of SGLT-2 inhibition versus placebo (and its 95% confidence interval [CI]). Intention-to-treat population data and analyses were used, wherever possible.

Risk of bias assessment

Risk of bias was assessed independently and in duplicate (AJR, AR, AK, AW) using the Cochrane Risk of Bias 2 (ROB2) tool, which assesses risk of bias in studies according to five domains: randomization process, deviations from the intended interventions, missing outcome data, measurement of the outcome, and selection of the reported result.

Search terms and strategy

MEDLINE Search strategy

1	randomized controlled trial.pt.
2	controlled clinical trial.pt.
3	randomized.ab.
4	placebo.ab.
5	clinical trials as topic.sh.
6	randomly.ab.
7	trial.ti.

8	1 or 2 or 3 or 4 or 5 or 6 or 7
9	exp animals/ not humans.sh.
10	8 not 9
11	exp Sodium-Glucose Transporter 2 Inhibitors/
12	sglt2.tw.
13	sglt-2.tw.
14	exp Sodium-Glucose Transporter 2/
15	sodium-glucose transporter$.tw.
16	sodium-glucose co-transporter$.tw.
17	sodium-glucose cotransporter$.tw.
18	(canagliflozin$ or dapagliflozin$ or empagliflozin$ or ertugliflozin$ or ipragliflozin$ or luseogliflozin$ or remogliflozin$ or sergliflozin$ or sotagliflozin$ or tofogliflozin$).tw.
19	11 or 12 or 13 or 14 or 15 or 16 or 17 or 18
20	10 and 19

EMBASE search terms and strategy

1	Randomized controlled trial/
2	Controlled clinical study/
3	random$.ti,ab.
4	randomization/
5	intermethod comparison/
6	placebo.ti,ab.
7	(compare or compared or comparison).ti.
8	((evaluated or evaluate or evaluating or assessed or assess) and (compare or compared or comparing or comparison)).ab.
9	(open adj label).ti,ab.
10	((double or single or doubly or singly) adj (blind or blinded or blindly)).ti,ab.
11	double blind procedure/
12	parallel group$1.ti,ab.
13	(crossover or cross over).ti,ab.
14	((assign$ or match or matched or allocation) adj5 (alternate or group$1 or intervention$1 or patient$1 or subject$1 or participant$1)).ti,ab.
15	(assigned or allocated).ti,ab.
16	(controlled adj7 (study or design or trial)).ti,ab.
17	(volunteer or volunteers).ti,ab.
18	human experiment/
19	trial.ti.
20	1 or 2 or 3 or 4 or 5 or 6 or 7 or 8 or 9 or 10 or 11 or 12 or 13 or 14 or 15 or 16 or 17 or 18 or 19

21	(random$ adj sampl$ adj7 ("cross section$" or questionnaire$1 or survey$ or database$1)).ti,ab. not (comparative study/ or controlled study/ or randomi?ed controlled.ti,ab. or randomly assigned.ti,ab.)
22	Cross-sectional study/ not (randomized controlled trial/ or controlled clinical study/ or controlled study/ or randomi?ed controlled.ti,ab. or control group$1.ti,ab.)
23	(((case adj control$) and random$) not randomi?ed controlled).ti,ab.
24	(Systematic review not (trial or study)).ti.
25	(nonrandom$ not random$).ti,ab.
26	"Random field$".ti,ab.
27	(random cluster adj3 sampl$).ti,ab.
28	(review.ab. and review.pt.) not trial.ti.
29	"we searched".ab. and (review.ti. or review.pt.)
30	"update review".ab.
31	(databases adj4 searched).ab.
32	(rat or rats or mouse or mice or swine or porcine or murine or sheep or lambs or pigs or piglets or rabbit or rabbits or cat or cats or dog or dogs or cattle or bovine or monkey or monkeys or trout or marmoset$1).ti. and animal experiment/
33	Animal experiment/ not (human experiment/ or human/)
34	21 or 22 or 23 or 24 or 25 or 26 or 27 or 28 or 29 or 30 or 31 or 32 or 33
35	20 not 34
36	exp Sodium-Glucose Transporter 2 Inhibitors/
37	sglt2.tw.
38	sglt-2.tw.
39	exp Sodium-Glucose Transporter 2/
40	sodium-glucose transporter$.tw.
41	sodium-glucose co-transporter$.tw.
42	sodium-glucose cotransporter$.tw.
43	(canagliflozin$ or dapagliflozin$ or empagliflozin$ or ertugliflozin$ or ipragliflozin$ or luseogliflozin$ or remogliflozin$ or sergliflozin$ or sotagliflozin$ or tofogliflozin$).tw.
44	36 or 37 or 38 or 39 or 40 or 41 or 42 or 43

Data from eligible trials not included in meta-analyses

InTandem3* (24) was identified by the systematic review but not included in meta-analyses as it was in a population with type 1 DM and therefore did not fit into one of the 3 pre-defined patient groups. Follow-up was also only 24 weeks precluding the reporting of a large number of relevant outcomes. Outcomes relevant to this meta-analysis are summarized in the table below:

	Sotagliflozin (n=699) Participants with events	Placebo (n=703) Participants with events
**Major cardiovascular event**	2	0
Myocardial infarction	1	0
Coronary revascularization	1	0
Stroke	0	0
**Hospitalization for heart failure or cardiovascular death**	0	0
**"Renal event"**	5	3
**Ketoacidosis**	21	4
**Severe hypoglycaemia**	21	17
**Amputation**	0	0
**Bone fracture**	4	5
**Urinary tract infection**	25	27
**Any death**	1	0

* 24 week trial in a population with type 1 DM with primary outcome of: glycated haemoglobin level <7.0% at week 24, with no episodes of severe hypoglycaemia or diabetic ketoacidosis after randomization. Kidney disease progression and acute kidney injury events data not available.

The Dapagliflozin in Respiratory Failure in Patients With COVID-19 (DARE-19) trial (25) was identified by the systematic review but not included in meta-analysis given the study population (hospitalized patients with COVID-19) and very short treatment and follow-up duration (30 days), limiting reporting of most relevant outcomes. Reported outcomes relevant to this meta-analysis are summarized below:

	Dapagliflozin (n=625) Participants with events	Placebo (n=625) Participants with events
**Any death**	41	54
**Acute kidney injury**	21	34
**Diabetic ketoacidosis**	2	0

Additional statistical methods

Meta-analysis method for rare outcomes

Inverse-variance-weighted averages of log hazard ratios/relative risks (RRs) were used to estimate the treatment effects in each patient group and overall. For the very rare outcome of Fournier's gangrene, the observed minus expected number of events (O-E) and its variance, V (calculated from the 2x2 contingency table) were used to calculate the one-step estimate of the odds ratio for individual trials. The sum of the O-E and V in the relevant trials were used to estimate the treatment effects in each patient group and overall for this rare outcome. For ketoacidosis, trials with zero events in one arm had a zero cell count correction applied (by adding 0.5 to arms with no events).

Adjustment factor for different definitions of % decline in estimated glomerular filtration rate (eGFR)

A sensitivity analysis was performed to assess how different definitions of kidney disease progression might have affected results. These analyses used data from CANVAS from which effect sizes for different definitions of the composite kidney disease progression outcome using a range of eGFR decline thresholds (≥57% [equivalent to a doubling of creatinine], ≥50%, and ≥40%) have been reported (26). Adjustment factors from CANVAS were estimated from the relative differences in the log RR and the associated standard errors for each % decline compared to a ≥40% eGFR decline. For trials that have only reported kidney disease progression using either of a ≥50% decline in eGFR or a doubling of creatinine, our sensitivity analysis then used these CANVAS adjustment factors to re-estimate RRs and 95%CIs for the hypothetical scenario that their kidney disease progression outcomes had used a ≥40% decline in eGFR.

References for Supplemental Methods

1. Page MJ, McKenzie JE, Bossuyt PM, Boutron I, Hoffmann TC, Mulrow CD, et al. The PRISMA 2020 statement: an updated guideline for reporting systematic reviews. BMJ. 2021;372:n71.
2. Higgins JPT, Thomas J, Chandler J, Cumpston M, Li T, Page MJ, Welch VA (editors). Cochrane Handbook for Systematic Reviews of Interventions version 6.2 (updated February 2021). Cochrane, 2021. Available from www.training.cochrane.org/handbook (accessed 27th April 2021).
3. McMurray JJV, Solomon SD, Inzucchi SE, Kober L, Kosiborod MN, Martinez FA, et al. Dapagliflozin in Patients with Heart Failure and Reduced Ejection Fraction. N Engl J Med. 2019;381(21):1995-2008.
4. Packer M, Anker SD, Butler J, Filippatos G, Pocock SJ, Carson P, et al. Cardiovascular and Renal Outcomes with Empagliflozin in Heart Failure. N Engl J Med. 2020;383(15):1413-24.
5. Bhatt DL, Szarek M, Steg PG, Cannon CP, Leiter LA, McGuire DK, et al. Sotagliflozin in Patients with Diabetes and Recent Worsening Heart Failure. N Engl J Med. 2021;384(2):117-28.
6. Zinman B, Wanner C, Lachin JM, Fitchett D, Bluhmki E, Hantel S, et al. Empagliflozin, Cardiovascular Outcomes, and Mortality in Type 2 Diabetes. N Engl J Med. 2015;373(22):2117-28.
7. Neal B, Perkovic V, Mahaffey KW, de Zeeuw D, Fulcher G, Erondu N, et al. Canagliflozin and Cardiovascular and Renal Events in Type 2 Diabetes. N Engl J Med. 2017;377(7):644-57.
8. Wiviott SD, Raz I, Bonaca MP, Mosenzon O, Kato ET, Cahn A, et al. Dapagliflozin and Cardiovascular Outcomes in Type 2 Diabetes. N Engl J Med. 2019;380(4):347-57.
9. Cannon CP, Pratley R, Dagogo-Jack S, Mancuso J, Huyck S, Masiukiewicz U, et al. Cardiovascular Outcomes with Ertugliflozin in Type 2 Diabetes. N Engl J Med. 2020;383(15):1425-35.
10. Heerspink HJL, Stefansson BV, Correa-Rotter R, Chertow GM, Greene T, Hou FF, et al. Dapagliflozin in Patients with Chronic Kidney Disease. N Engl J Med. 2020;383(15):1436-46.
11. Bhatt DL, Szarek M, Pitt B, Cannon CP, Leiter LA, McGuire DK, et al. Sotagliflozin in Patients with Diabetes and Chronic Kidney Disease. N Engl J Med. 2021;384(2):129-39.
12. Perkovic V, Jardine MJ, Neal B, Bompoint S, Heerspink HJL, Charytan DM, et al. Canagliflozin and Renal Outcomes in Type 2 Diabetes and Nephropathy. N Engl J Med. 2019;380(24):2295-306.
13. Anker SD, Butler J, Filippatos G, Ferreira JP, Bocchi E, Bohm M, et al. Empagliflozin in Heart Failure with a Preserved Ejection Fraction. N Engl J Med. 2021.
14. Zannad F, Ferreira JP, Pocock SJ, Zeller C, Anker SD, Butler J, et al. Cardiac and Kidney Benefits of Empagliflozin in Heart Failure Across the Spectrum of Kidney Function: Insights From EMPEROR-Reduced. Circulation. 2021;143(4):310-21.
15. Anker SD, Butler J, Filippatos G, Khan MS, Marx N, Lam CSP, et al. Effect of Empagliflozin on Cardiovascular and Renal Outcomes in Patients With Heart Failure by Baseline Diabetes Status: Results From the EMPEROR-Reduced Trial. Circulation. 2021;143(4):337-49.
16. Inzucchi SE, Iliev H, Pfarr E, Zinman B. Empagliflozin and Assessment of Lower-Limb Amputations in the EMPA-REG OUTCOME Trial. Diabetes Care. 2018;41(1):e4-e5.
17. Mahaffey KW, Neal B, Perkovic V, de Zeeuw D, Fulcher G, Erondu N, et al. Canagliflozin for Primary and Secondary Prevention of Cardiovascular Events: Results From the CANVAS Program (Canagliflozin Cardiovascular Assessment Study). Circulation. 2018;137(4):323-34.
18. Mahaffey KW, Jardine MJ, Bompoint S, Cannon CP, Neal B, Heerspink HJL, et al. Canagliflozin and Cardiovascular and Renal Outcomes in Type 2 Diabetes Mellitus and Chronic Kidney Disease in Primary and Secondary Cardiovascular Prevention Groups. Circulation. 2019;140(9):739-50.
19. Neuen BL, Ohkuma T, Neal B, Matthews DR, de Zeeuw D, Mahaffey KW, et al. Relative and Absolute Risk Reductions in Cardiovascular and Kidney Outcomes With Canagliflozin Across KDIGO Risk Categories: Findings From the CANVAS Program. Am J Kidney Dis. 2021;77(1):23-34 e1.
20. Wheeler DC, Stefansson BV, Jongs N, Chertow GM, Greene T, Hou FF, et al. Effects of dapagliflozin on major adverse kidney and cardiovascular events in patients with diabetic and non-diabetic chronic kidney disease: a prespecified analysis from the DAPA-CKD trial. Lancet Diabetes Endocrinol. 2021;9(1):22-31.
21. Heerspink HJL, Sjostrom CD, Jongs N, Chertow GM, Kosiborod M, Hou FF, et al. Effects of dapagliflozin on mortality in patients with chronic kidney disease: a pre-specified analysis from the DAPA-CKD randomized controlled trial. Eur Heart J. 2021;42(13):1216-27.
22. Herrington WG, Preiss D, Haynes R, von Eynatten M, Staplin N, Hauske SJ, et al. The potential for improving cardio-renal outcomes by sodium-glucose co-transporter-2 inhibition in people with chronic kidney disease: a rationale for the EMPA-KIDNEY study. Clin Kidney J. 2018;11(6):749-61.
23. Packer M, Butler J, Zannad F, Pocock SJ, Filippatos G, Ferreira JP, et al. Empagliflozin and Major Renal Outcomes in Heart Failure. N Engl J Med. 2021.
24. Garg SK, Henry RR, Banks P, Buse JB, Davies MJ, Fulcher GR, et al. Effects of Sotagliflozin Added to Insulin in Patients with Type 1 Diabetes. N Engl J Med. 2017;377(24):2337-48.

25. Kosiborod MN, Esterline R, Furtado RHM, Oscarsson J, Gasparyan SB, Koch GG, et al. Dapagliflozin in patients with cardiometabolic risk factors hospitalised with COVID-19 (DARE-19): a randomised, double-blind, placebo-controlled, phase 3 trial. Lancet Diabetes Endocrinol. 2021;9(9):586-94.
26. Oshima M, Neal B, Toyama T, Ohkuma T, Li Q, de Zeeuw D, et al. Different eGFR Decline Thresholds and Renal Effects of Canagliflozin: Data from the CANVAS Program. J Am Soc Nephrol. 2020;31(10):2446-56.

Supplemental table 1: Quality assessments

Unique ID	Study ID	Experimental	Comparator	D1	D2	D3	D4	D5	Overall
1	CREDENCE	Canagliflozin	Placebo	+	+	+	+	+	+
2	CANVAS	Canagliflozin	Placebo	+	+	+	+	+	+
3	DAPA-CKD	Dapagliflozin	Placebo	+	+	+	+	+	+
4	DAPA-HF	Dapagliflozin	Placebo	+	+	+	+	+	+
5	DECLARE-TIMI 58	Dapagliflozin	Placebo	+	+	+	+	+	+
6	EMPA-REG OUTCOME	Empagliflozin	Placebo	+	+	+	+	+	+
7	EMPEROR-PRESERVED	Empagliflozin	Placebo	+	+	+	+	+	+
8	EMPEROR-REDUCED	Empagliflozin	Placebo	+	+	+	+	+	+
9	InTANDEM3	Sotagliflozin	Placebo	+	+	+	+	+	+
10	SCORED	Sotagliflozin	Placebo	+	+	+	+	+	+
11	SOLOIST-WHF	Sotagliflozin	Placebo	+	+	+	+	+	+
12	VERTIS CV	Ertugliflozin	Placebo	+	+	+	+	+	+

+ Low risk
! Some concerns
- High risk

D1 Randomization process
D2 Deviations from the intended interventions
D3 Missing outcome data
D4 Measurement of the outcome
D5 Selection of the reported result

Supplemental Table 2: Predicted absolute benefits and harms of SGLT-2 inhibitors per 1000 patient years of treatment, by patient group (sensitivity analysis using patient group-specific relative risks)

	Absolute rates and effects per 1000 patient years									
	STABLE HEART FAILURE				RECENTLY HOSPITALIZED FOR WORSENING HEART FAILURE		TYPE 2 DIABETES MELLITUS AT HIGH ATHEROSCLEROTIC CARDIOVASCULAR RISK		ALBUMINURIC CHRONIC KIDNEY DISEASE	
	REDUCED EJECTION FRACTION		PRESERVED EJECTION FRACTION							
	Event rate	Events avoided/ caused (SE) in SGLT-2i arms	Event rate	Events avoided/ caused (SE) in SGLT-2i arms	Event rate	Events avoided/ caused (SE) in SGLT-2i arms	Event rate	Events avoided/ caused (SE) in SGLT-2i arms	Event rate	Events avoided/ caused (SE) in SGLT-2i arms
**Efficacy outcomes**										
Hospitalization for heart failure	123	-38 (4)	60	-18 (2)	639	-196 (20)	10	-3 (0.4)	20	-7 (0.9)
Myocardial infarction	-	-	-	-	-	-	15	-1 (0.6)	9	-2 (0.7)
Cardiovascular death	80	-10 (4)	38	-5 (2)	125	-16 (6)	13	-2 (0.5)	21	-3 (1)
Kidney disease progression	20	-4 (2)	22	-5 (2)	-	-	9	-4 (0.4)	49	-19 (2)
Acute kidney injury	19	-6 (2)	-	-	59	-20 (5)	4	-1 (0.2)	15	-3 (1)
**Safety outcomes**										
Ketoacidosis	-	-	-	-	-	-	0.2	0.3 (0.2)	0.3	0.4 (0.2)
Amputation	4	-0.1 (0.8)	4	-0.1 (0.7)	2	-0.0 (0.5)	4	1 (0.4)	9	0.1 (1.2)

Patient group specific absolute effects estimated by applying the observed patient group-specific relative risk to the average event rate in the placebo arms (first event only). For the heart failure patient groups the placebo event rates were estimated separately for trials of stable heart failure with reduced ejection fraction (i.e. EMPEROR-REDUCED & DAPA-HF) versus stable heart failure with preserved ejection fraction (i.e. EMPEROR-PRESERVED) versus recent hospitalization for heart failure (i.e. SOLOIST-WHF). Standard errors (SE) in the numbers of events avoided or caused estimated from uncertainty in the relative risks. Kidney disease progression definitions were as reported by trials (i.e. not uniformly adjusted to a ≥40% eGFR decline). There were too few ketoacidosis events to estimate absolute effects in heart failure patient groups.

Supplemental Table 3: Predicted absolute benefits and harms of SGLT-2 inhibitors per 1000 patient years of treatment in A) STABLE HEART FAILURE and B) ALBUMINURIC CHRONIC KIDNEY DISEASE, by diabetes status

A) STABLE HEART FAILURE

	Absolute rates and effects per 1000 patient years							
	REDUCED EJECTION FRACTION				PRESERVED EJECTION FRACTION			
	Type 2 diabetes mellitus		No diabetes		Type 2 diabetes mellitus		No diabetes	
	Event rate	Events avoided/ caused (SE) in SGLT-2i arms	Event rate	Events avoided/ caused (SE) in SGLT-2i arms	Event rate	Events avoided/ caused (SE) in SGLT-2i arms	Event rate	Events avoided/ caused (SE) in SGLT-2i arms
**Efficacy outcomes**								
Hospitalization for heart failure or cardiovascular death	204	-48 (4)	141	-33 (3)	91	-21 (2)	66	-15 (1)
Kidney disease progression	42	-15 (1)	20	-7 (0.5)	23	-8 (0.6)	12	-4 (0.3)
**Safety outcomes**								
Ketoacidosis	-	-	-	-	2	2 (0.6)	-	-
Amputation	8	1 (0.6)	-	-	2	0.3 (0.1)	-	-

Patient group specific absolute effects estimated by applying the overall relative risk to the average event rate in the placebo arms (first event only). Standard errors (SE) in the numbers of events avoided or caused estimated from uncertainty in the relative risks. Kidney disease progression definitions were as reported by trials (i.e. not uniformly adjusted to a ≥40% eGFR decline). There were too few ketoacidosis events to estimate absolute effects in the heart failure patient group. There were too few ketoacidosis and amputation events to estimate absolute effects amongst patients without type 2 diabetes mellitus. Rates of amputation events among patients with type 2 diabetes mellitus not available in stable heart failure with preserved ejection fraction.

B) ALBUMINURIC CHRONIC KIDNEY DISEASE

	Absolute rates and effects per 1000 patient years			
	Type 2 diabetes mellitus		No diabetes	
	Event rate	Events avoided/ caused (SE) in SGLT-2i arms	Event rate	Events avoided/ caused (SE) in SGLT-2i arms
**Efficacy outcomes**				
Hospitalization for heart failure or cardiovascular death	42	-10 (0.8)	13	-3 (0.2)
Kidney disease progression	48	-17 (1)	53	-19 (1)
**Safety outcomes**				
Ketoacidosis	0.4	0.4 (0.1)	-	-
Amputation	11	2 (0.8)	-	-

Patient group specific absolute effects estimated by applying the overall relative risk to the average event rate in the placebo arms (first event only). Standard errors (SE) in the numbers of events avoided or caused estimated from uncertainty in the relative risks. Kidney disease progression definitions were as reported by trials (i.e. not uniformly adjusted to a ≥40% eGFR decline). There were too few ketoacidosis and amputation events to estimate absolute effects amongst patients without type 2 diabetes mellitus.

Supplemental figure 1: Effect of SGLT2 inhibitors on (a) HOSPITALIZATION FOR HEART FAILURE OR CARDIOVASCULAR DEATH and (b) KIDNEY DISEASE PROGRESSION, by diabetes status and by trial

	HOSPITALIZATION FOR HEART FAILURE OR CARDIOVASCULAR DEATH				KIDNEY DISEASE PROGRESSION			
	Number of events/ participants	Rate per 1000 patient years SGLT2i	Rate per 1000 patient years Placebo	Relative risk (95% CI)	Number of events/ participants	Rate per 1000 patient years SGLT2i	Rate per 1000 patient years Placebo	Relative risk (95% CI)
**Heart failure**								
DAPA−HF (diabetes)	486/2139	132	168	0.75 (0.63−0.90)	NA/2139	−	−	0.73 (0.39−1.34)
DAPA−HF (no diabetes)	402/2605	87	116	0.73 (0.60−0.88)	NA/2603	−	−	0.67 (0.30−1.49)
EMPEROR−REDUCED (diabetes)	465/1856	177	246	0.72 (0.60−0.87)	61/1856	23	42	0.53 (0.31−0.90)
EMPEROR−REDUCED (no diabetes)	358/1874	139	176	0.78 (0.64−0.97)	27/1874	8.3	20	0.42 (0.19−0.97)
SOLOIST−WHF	NA/1222	−	−	0.71 (0.56−0.89)				
EMPEROR−PRESERVED (diabetes)	530/2938	75	90	0.79 (0.67−0.94)	149/2938	23	23	1.00 (0.72−1.38)
EMPEROR−PRESERVED (no diabetes)	396/3050	53	66	0.78 (0.64−0.95)	71/3050	9.9	12	0.87 (0.54−1.38)
**All heart failure trials**	**2637/15684**			**0.76 (0.70−0.82)**	**375/14460**			**0.78 (0.64−0.96)**
**Type 2 DM at high ASCVD risk**								
EMPA−REG OUTCOME	463/7020	20	30	0.66 (0.55−0.79)	152/6968	6.3	12	0.54 (0.40−0.75)
CANVAS Program	652/10142	16	21	0.78 (0.67−0.91)	249/10142	5.5	9	0.60 (0.47−0.77)
DECLARE−TIMI58	913/17160	12	15	0.83 (0.73−0.95)	365/17160	3.7	7	0.53 (0.43−0.66)
VERTIS CV	694/8246	23	27	0.88 (0.75−1.03)	283/8246	9	12	0.81 (0.63−1.04)
**All type 2 DM at high ASCVD risk trials**	**2722/42568**			**0.80 (0.74−0.86)**	**1049/42516**			**0.61 (0.54−0.69)**
**Chronic kidney disease**								
CREDENCE	432/4401	32	45	0.69 (0.57−0.83)	377/4401	27	40	0.66 (0.53−0.81)
DAPA−CKD (diabetes)	204/2906	27	38	0.70 (0.53−0.92)	276/2906	35	60	0.57 (0.45−0.73)
DAPA−CKD (no diabetes)	34/1398	11	13	0.79 (0.40−1.55)	109/1398	29	53	0.51 (0.34−0.75)
SCORED	640/10584	40	51	0.77 (0.66−0.91)	89/10584	5	7	0.71 (0.46−1.08)
**All chronic kidney disease trials**	**1310/19289**			**0.73 (0.65−0.82)**	**851/19289**			**0.62 (0.54−0.71)**
**OVERALL**	**6669/77541**			**0.76 (0.72−0.80)**	**2275/76265**			**0.64 (0.59−0.70)**

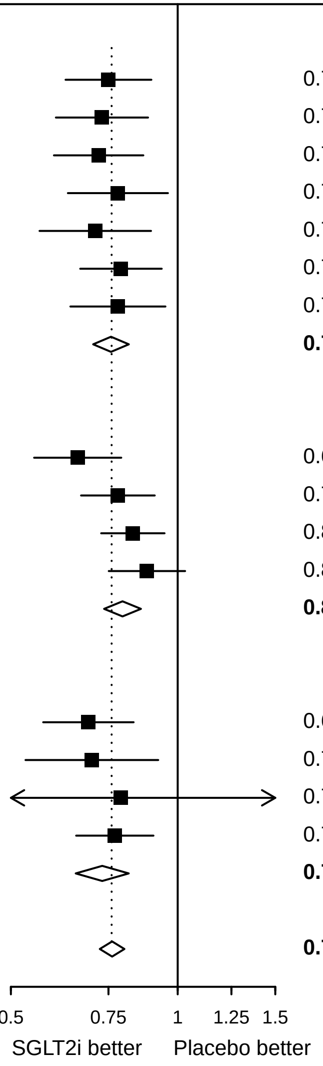

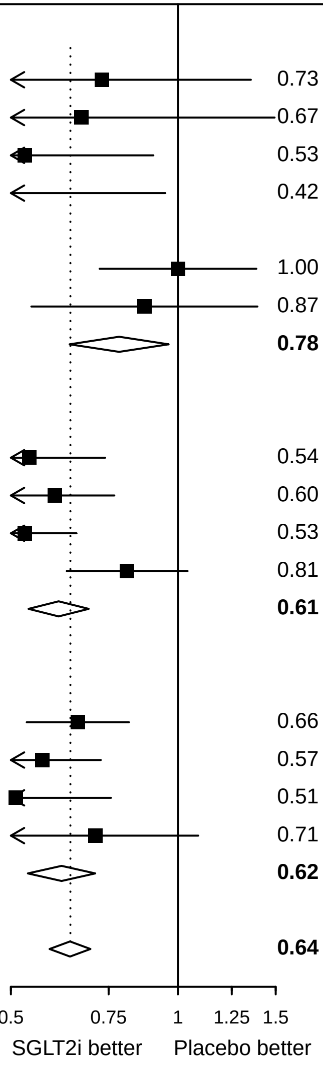

DM = diabetes mellitus. ASCVD = atherosclerotic cardiovascular disease. For hospitalization for heart failure or cardiovascular death, results for DAPA−HF include urgent visits for heart failure. Number of kidney disease progression events by diabetes subgroup not available (NA) for DAPA−HF. Kidney disease progression outcomes are not adjusted for different definitions: see footnote to Figure 3 for more details

Supplemental figure 2: Effect of SGLT2 inhibitors on HOSPITALIZATION FOR HEART FAILURE, by patient group and by trial

	Number of events/ participants	Rate per 1000 patient years SGLT-2i	Rate per 1000 patient years Placebo	Relative risk (95% CI)
**Heart failure**				
DAPA-HF	549/4744	69	98	0.70 (0.59-0.83)
EMPEROR-REDUCED	588/3730	107	155	0.69 (0.59-0.81)
SOLOIST-WHF	491/1222	404	639	0.64 (0.49-0.83)
EMPEROR-PRESERVED	611/5988	43	60	0.71 (0.60-0.83)
**All heart failure trials**	**2239/15684**			**0.69 (0.63-0.76)**
**Type 2 DM at high ASCVD risk**				
EMPA-REG OUTCOME	221/7020	9.4	15	0.65 (0.50-0.85)
CANVAS Program	243/10142	5.5	8.7	0.67 (0.52-0.87)
DECLARE-TIMI58	498/17160	6.2	8.5	0.73 (0.61-0.88)
VERTIS CV	238/8246	7	11	0.70 (0.54-0.90)
**All type 2 DM at high ASCVD risk trials**	**1200/42568**			**0.70 (0.62-0.78)**
**Chronic kidney disease**				
CREDENCE	230/4401	16	25	0.61 (0.47-0.80)
DAPA-CKD	108/4304	7.2	14	0.52 (0.35-0.77)
SCORED	605/10584	35	51	0.67 (0.55-0.82)
**All chronic kidney disease trials**	**943/19289**			**0.63 (0.54-0.73)**
**OVERALL**	**4382/77541**			**0.68 (0.64-0.73)**

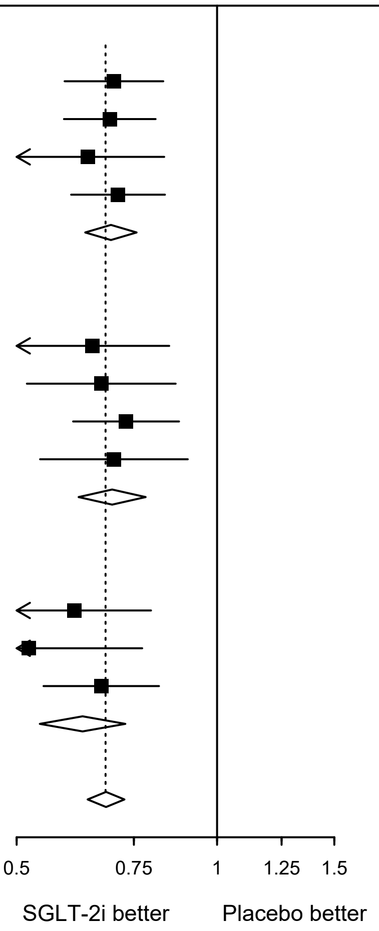

Heterogeneity between trials within patient groups:
Heart failure p=0.93;
Type 2 DM at high ASCVD risk p=0.9;
Chronic kidney disease: p=0.52

Heterogeneity between 3 patient groups: p=0.49

DM = diabetes mellitus. ASCVD = atherosclerotic cardiovascular disease. Results are based on time to first event analyses and exclude urgent visits for heart failure, wherever possible. Event rates estimated from number of events and follow-up duration for SCORED.

Supplemental figure 3: Effect of SGLT2 inhibition on (a) MYOCARDIAL INFARCTION and (b) STROKE, by patient group

MYOCARDIAL INFARCTION

	Number of events/ participants	Rate per 1000 patient years SGLT2i	Rate per 1000 patient years Placebo	Relative risk (95% CI)
**Type 2 DM at high ASCVD risk**				
EMPA−REG OUTCOME	349/7020	17	19	0.87 (0.70−1.09)
CANVAS Program	421/10142	11	13	0.89 (0.73−1.09)
DECLARE−TIMI58	834/17160	12	13	0.89 (0.77−1.01)
VERTIS CV	488/8246	18	17	1.04 (0.86−1.26)
**All type 2 DM at high ASCVD risk trials**	**2092/42568**			**0.92 (0.84−1.00)**
**Chronic kidney disease**				
CREDENCE	178/4401	15	17	0.86 (0.64−1.16)
SCORED	NA/10584	−	−	0.68 (0.52−0.89)
**All chronic kidney disease trials**	**178/14985**			**0.76 (0.62−0.92)**
**OVERALL**	**2270/57553**			**0.89 (0.82−0.96)**

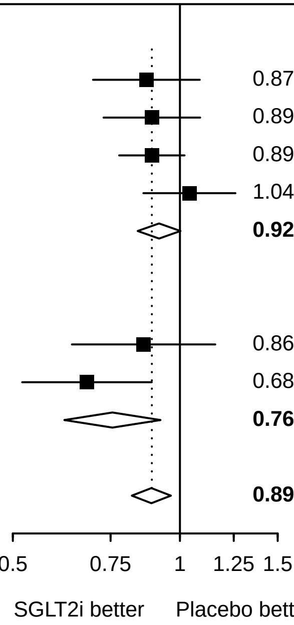

Heterogeneity between trials within patient groups:
Type 2 DM at high ASCVD risk p=0.54;
Chronic kidney disease: p=0.25

Heterogeneity between 2 patient groups: p=0.08

STROKE

	Number of events/ participants	Rate per 1000 patient years SGLT2i	Rate per 1000 patient years Placebo	Relative risk (95% CI)
**Type 2 DM at high ASCVD risk**				
EMPA−REG OUTCOME	233/7020	12	11	1.18 (0.89−1.56)
CANVAS Program	309/10142	7.9	9.6	0.87 (0.69−1.09)
DECLARE−TIMI58	466/17160	6.9	6.8	1.01 (0.84−1.21)
VERTIS CV	272/8246	10	9	1.06 (0.82−1.37)
**All type 2 DM at high ASCVD risk trials**	**1280/42568**			**1.01 (0.90−1.13)**
**Chronic kidney disease**				
CREDENCE	142/4401	11	14	0.77 (0.55−1.08)
SCORED	NA/10584	−	−	0.66 (0.48−0.91)
**All chronic kidney disease trials**	**142/14985**			**0.71 (0.56−0.90)**
**OVERALL**	**1422/57553**			**0.94 (0.85−1.04)**

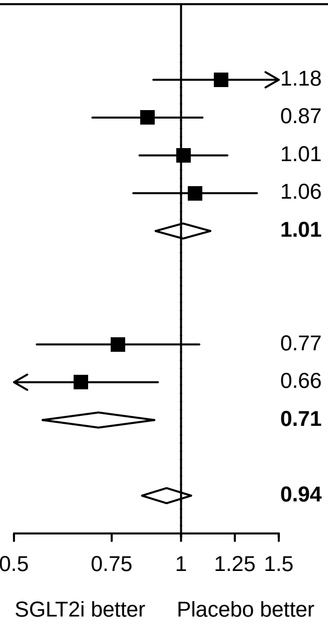

Heterogeneity between trials within patient groups:
Type 2 DM at high ASCVD risk p=0.4;
Chronic kidney disease: p=0.52

Heterogeneity between 2 patient groups: p=0.01

DM = diabetes mellitus. ASCVD = atherosclerotic cardiovascular disease. Results unavailable for the heart failure patient group trials. Numbers of events unavailable for SCORED. EMPA−REG OUTCOME excluded silent myocardial infarction from its myocardial infarction outcome. DECLARE−TIMI58 included only ischaemic stroke in the presented stroke outcome.

Supplemental figure 4: Effect of SGLT2 inhibitors on DEATH from ANY CAUSE, by patient group

	Number of events/ participants	Rate per 1000 patient years SGLT-2i	Rate per 1000 patient years Placebo	Relative risk (95% CI)
**Heart failure**				
DAPA-HF	605/4744	79	95	0.83 (0.71-0.97)
EMPEROR-REDUCED	515/3730	101	107	0.92 (0.77-1.10)
SOLOIST-WHF	141/1222	135	163	0.82 (0.59-1.14)
EMPEROR-PRESERVED	849/5988	66	67	1.00 (0.87-1.15)
**All heart failure trials**	**2110/15684**			**0.91 (0.84-1.00)**
**Type 2 DM at high ASCVD risk**				
EMPA-REG OUTCOME	463/7020	19	29	0.68 (0.57-0.82)
CANVAS Program	681/10142	17	20	0.87 (0.74-1.01)
DECLARE-TIMI58	1099/17160	15	16	0.93 (0.82-1.04)
VERTIS CV	727/8246	24	26	0.93 (0.80-1.08)
**All type 2 DM at high ASCVD risk trials**	**2970/42568**			**0.87 (0.81-0.94)**
**Chronic kidney disease**				
CREDENCE	369/4401	29	35	0.83 (0.68-1.02)
DAPA-CKD	247/4304	22	31	0.69 (0.53-0.88)
SCORED	492/10584	35	35	0.99 (0.83-1.18)
**All chronic kidney disease trials**	**1108/19289**			**0.86 (0.77-0.97)**
**OVERALL**	**6188/77541**			**0.88 (0.84-0.93)**

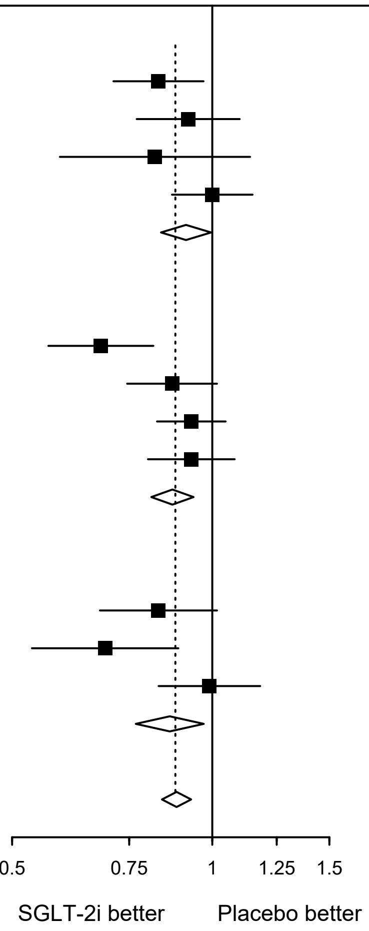

Heterogeneity between trials within patient groups:
Heart failure p=0.32;
Type 2 DM at high ASCVD risk p=0.03;
Chronic kidney disease: p=0.06

Heterogeneity between 3 patient groups: p=0.65

DM = diabetes mellitus. ASCVD = atherosclerotic cardiovascular disease.

Supplemental figure 5: Effect of SGLT-2 inhibitors on KIDNEY DISEASE PROGRESSION, by patient group and by trial, before and after applying adjustment factor for the different definitions of percent decline in eGFR

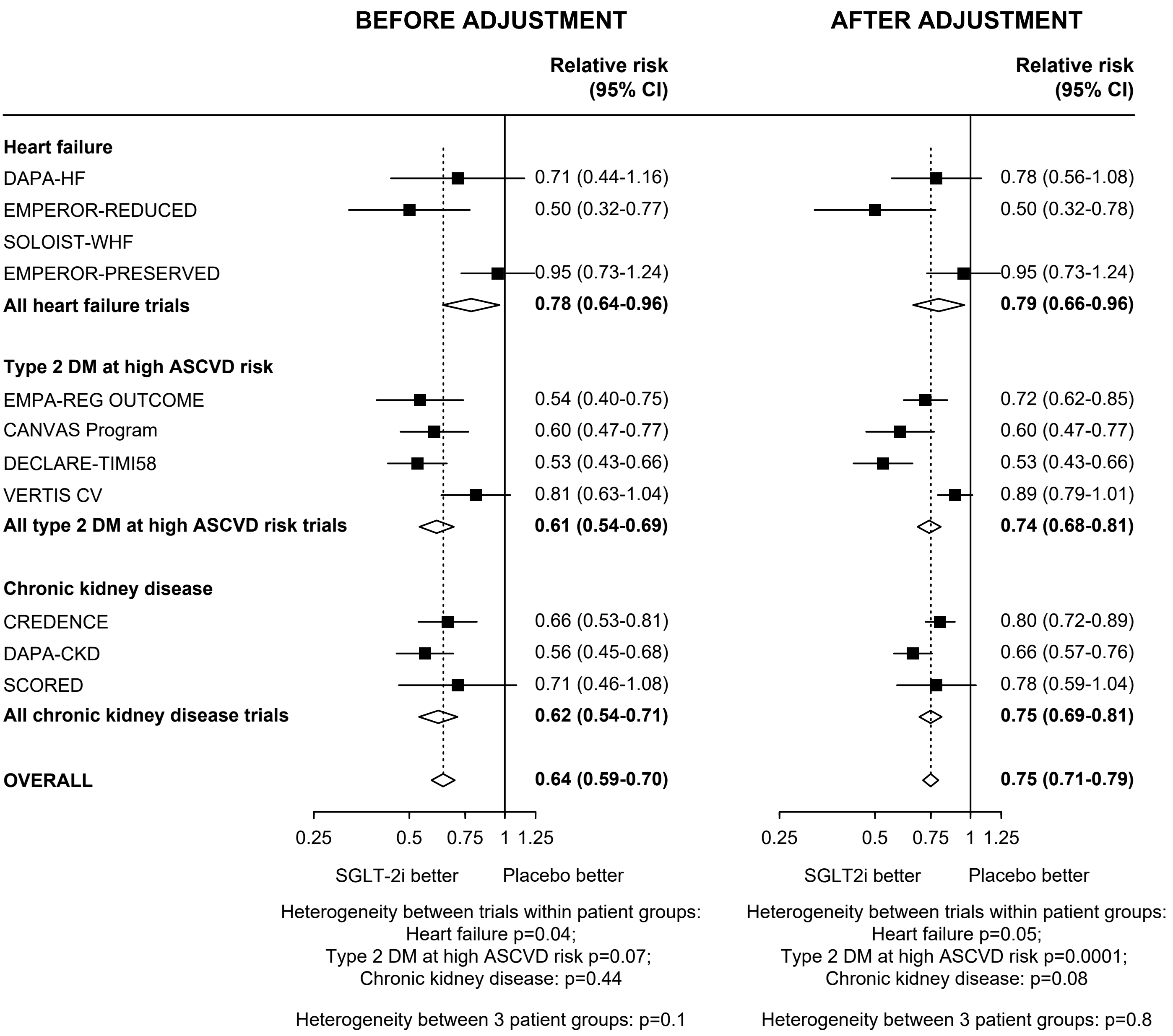

DM = diabetes mellitus. ASCVD = atherosclerotic cardiovascular disease. Kidney Disease Progression was generally defined as death from renal causes, commencement of renal replacement therapy, or a % decline in eGFR from baseline. The following trials used a ≥40% decline in eGFR: EMPEROR-REDUCED, EMPEROR-PRESERVED, CANVAS Program, DECLARE-TIMI58. The following trials used a ≥50% decline in eGFR: DAPA-HF, DAPA-CKD, SCORED. The following trials used a ≥57% decline in eGFR: EMPA-REG OUTCOME, VERTIS CV, CREDENCE. Results for kidney disease progression unavailable for SOLOIST-WHF. EMPA-REG OUTCOME population restricted to those that received at least one dose of study treatment. Effect sizes for different eGFR decline thresholds reported by CANVAS were used to calculate adjustment factors to allow relative risks and 95% confidence intervals to be re-estimated for the hypothetical scenario all trials had used a ≥40% decline in eGFR in the definition of their kidney disease progression composite outcome (see supplemental methods for full details).

Supplemental figure 6: Effect of SGLT2 inhibitors on (a) KETOACIDOSIS and (b) SEVERE HYPOGLYCAEMIA, by patient group and by trial

	KETOACIDOSIS				SEVERE HYPOGLYCAEMIA			
	Number of events/ participants	Rate per 1000 patient years SGLT2i	Rate per 1000 patient years Placebo	Relative risk (95% CI)	Number of events/ participants	Rate per 1000 patient years SGLT2i	Rate per 1000 patient years Placebo	Relative risk (95% CI)
**Heart failure**								
DAPA−HF	3/4736	0.8	0	6.00 (0.30−119.72)	8/4736	1.1	1.1	1.00 (0.25−3.99)
EMPEROR−REDUCED	0/3726	0	0		13/3730	2.4	2.8	0.86 (0.29−2.55)
SOLOIST−WHF	6/1216	4.4	8.7	0.50 (0.09−2.75)	11/1216	20	4.4	4.54 (0.99−20.95)
EMPEROR−PRESERVED	9/5985	0.6	0.8	0.80 (0.21−2.97)	151/5985	11	12	0.93 (0.68−1.28)
**All heart failure trials**	**18/11937**			**0.85 (0.32−2.27)**	**183/15667**			**0.99 (0.74−1.32)**
**Type 2 DM at high ASCVD risk**								
EMPA−REG OUTCOME	5/7020	0.3	0.1	1.99 (0.22−17.80)	99/7020	4.3	5	0.84 (0.56−1.26)
CANVAS Program	18/10142	0.6	0.3	2.16 (0.69−6.80)				
DECLARE−TIMI58	39/17143	0.7	0.3	2.18 (1.10−4.30)	141/17143	1.6	2.3	0.68 (0.49−0.95)
VERTIS CV	21/8238	1.2	0.2	4.75 (1.11−20.37)	446/8238	17	20	0.88 (0.73−1.06)
**All type 2 DM at high ASCVD risk trials**	**83/42543**			**2.40 (1.41−4.06)**	**686/32401**			**0.83 (0.71−0.96)**
**Chronic kidney disease**								
CREDENCE	12/4397	2.2	0.2	10.80 (1.39−83.65)	465/4397	44	49	0.92 (0.77−1.11)
DAPA−CKD	2/4298	0	0.4	0.25 (0.01−5.54)	42/4298	2.7	5.4	0.50 (0.26−0.95)
SCORED	44/10577	4.3	2	2.14 (1.14−4.03)	108/10577	7.5	7.8	0.96 (0.66−1.40)
**All chronic kidney disease trials**	**58/19272**			**2.27 (1.25−4.10)**	**615/19272**			**0.89 (0.76−1.05)**
**OVERALL**	**159/73752**			**2.03 (1.41−2.93)**	**1484/67340**			**0.87 (0.79−0.97)**

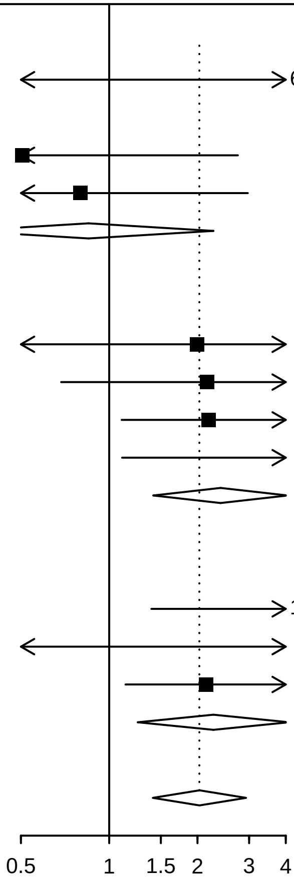

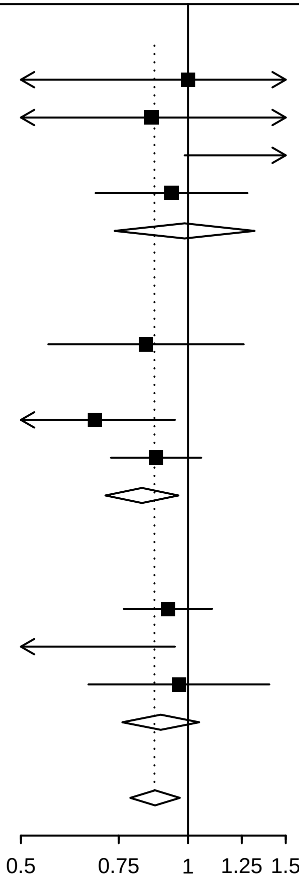

Ketoacidosis:

Heterogeneity between trials within patient groups:
Heart failure p=0.37;
Type 2 DM at high ASCVD risk p=0.81;
Chronic kidney disease: p=0.12

Heterogeneity between 3 patient groups: p=0.17

Severe hypoglycaemia:

Heterogeneity between trials within patient groups:
Heart failure p=0.26;
Type 2 DM at high ASCVD risk p=0.42;
Chronic kidney disease: p=0.18

Heterogeneity between 3 patient groups: p=0.53

DM = diabetes mellitus. ASCVD = atherosclerotic cardiovascular disease. Some studies limited analyses to a safety population. Treatment effects for ketoacidosis estimated from numbers of events for several trials (DAPA−HF, EMPEROR−REDUCED, SOLOIST−WHF, EMPEROR−PRESERVED, EMPA−REG OUTCOME, VERTIS CV, DAPA−CKD and SCORED). Where unreported, event rates for ketoacidosis are estimated from median follow−up and other reported information (DAPA−HF, SOLOIST−WHF, EMPEROR−PRESERVED, EMPA−REG OUTCOME, DECLARE−TIMI58, VERTIS CV, DAPA−CKD and SCORED). Treatment effects for severe hypoglycaemia estimated from numbers of events for several trials (DAPA−HF, EMPEROR−REDUCED, SOLOIST−WHF, EMPEROR−PRESERVED, CANVAS Program, VERTIS CV, DAPA−CKD and SCORED). Where unreported, event rates for severe hypoglycaemia are estimated from median follow−up and other reported information (DAPA−HF, EMPEROR−REDUCED, SOLOIST−WHF, EMPEROR−PRESERVED, EMPA−REG OUTCOME, DECLARE−TIMI58, VERTIS CV, DAPA−CKD and SCORED). EMPEROR−PRESERVED data on severe hypoglycaemia unavailable, so data on all hypoglycaemia events are shown.

Supplemental figure 7: Effect of SGLT2 inhibitors on (a) LOWER LIMB AMPUTATION and (b) BONE FRACTURE, by patient group and by trial

LOWER LIMB AMPUTATION

	Number of events/ participants	Rate per 1000 patient years SGLT2i	Rate per 1000 patient years Placebo	Relative risk (95% CI)
**Heart failure**				
DAPA−HF	25/4736	3.6	3.3	1.08 (0.50−2.37)
EMPEROR−REDUCED	23/3726	5.2	4	1.30 (0.57−2.96)
SOLOIST−WHF	5/1216	8.8	2.2	4.04 (0.45−36.04)
EMPEROR−PRESERVED	39/5985	2.4	3.5	0.69 (0.37−1.31)
**All heart failure trials**	**92/15663**			**0.98 (0.65−1.49)**
**Type 2 DM at high ASCVD risk**				
EMPA−REG OUTCOME	131/7020	6.5	6.5	1.00 (0.70−1.44)
CANVAS Program	187/10142	6.3	3.4	1.97 (1.41−2.75)
DECLARE−TIMI58	236/17143	3.4	3.1	1.09 (0.84−1.40)
VERTIS CV	156/8238	6.7	5.5	1.23 (0.87−1.74)
**All type 2 DM at high ASCVD risk trials**	**710/42543**			**1.25 (1.07−1.47)**
**Chronic kidney disease**				
CREDENCE	133/4397	12	11	1.11 (0.79−1.56)
DAPA−CKD	74/4298	6.8	7.6	0.90 (0.57−1.41)
SCORED	65/10577	4.5	4.7	0.97 (0.60−1.57)
**All chronic kidney disease trials**	**272/19272**			**1.01 (0.80−1.28)**
**OVERALL, excluding CANVAS**	**848/61351**			**1.06 (0.93−1.21)**
**OVERALL**	**1074/77478**			**1.16 (1.02−1.31)**

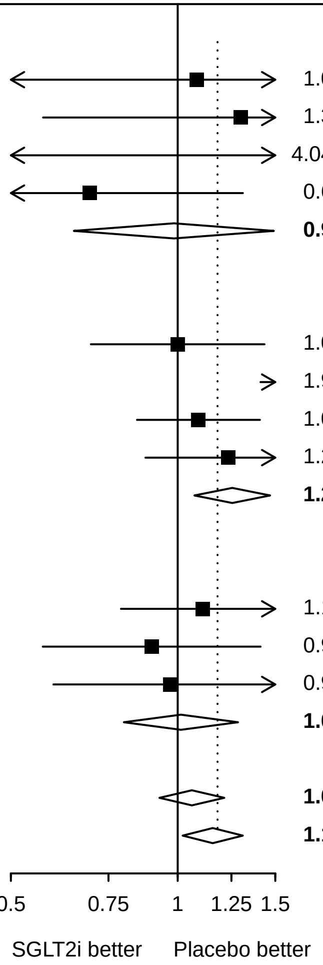

Heterogeneity between trials within patient groups:
Heart failure p=0.35;
Type 2 DM at high ASCVD risk p=0.02;
Chronic kidney disease: p=0.75

Heterogeneity between 3 patient groups: p=0.25

BONE FRACTURE

	Number of events/ participants	Rate per 1000 patient years SGLT2i	Rate per 1000 patient years Placebo	Relative risk (95% CI)
**Heart failure**				
DAPA−HF	99/4736	14	14	0.98 (0.66−1.45)
EMPEROR−REDUCED	87/3726	18	17	1.07 (0.71−1.62)
SOLOIST−WHF	21/1216	26	20	1.35 (0.57−3.17)
EMPEROR−PRESERVED	260/5985	20	19	1.06 (0.84−1.35)
**All heart failure trials**	**467/15663**			**1.06 (0.88−1.26)**
**Type 2 DM at high ASCVD risk**				
EMPA−REG OUTCOME	270/7020	12	13	0.94 (0.73−1.21)
CANVAS Program	496/10142	15	12	1.26 (1.04−1.52)
DECLARE−TIMI58	897/17143	13	12	1.04 (0.91−1.18)
VERTIS CV	299/8238	12	12	1.02 (0.81−1.30)
**All type 2 DM at high ASCVD risk trials**	**1962/42543**			**1.07 (0.98−1.17)**
**Chronic kidney disease**				
CREDENCE	135/4397	12	12	0.98 (0.70−1.37)
DAPA−CKD	154/4298	16	13	1.23 (0.90−1.68)
SCORED	228/10577	16	17	0.95 (0.73−1.23)
**All chronic kidney disease trials**	**517/19272**			**1.03 (0.87−1.23)**
**OVERALL, excluding CANVAS**	**2190/61351**			**1.03 (0.95−1.12)**
**OVERALL**	**2946/77478**			**1.06 (0.99−1.14)**

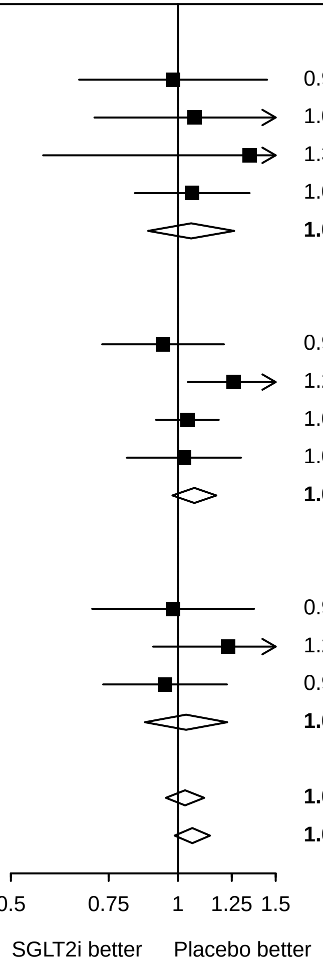

Heterogeneity between trials within patient groups:
Heart failure p=0.93;
Type 2 DM at high ASCVD risk p=0.24;
Chronic kidney disease: p=0.42

Heterogeneity between 3 patient groups: p=0.94

DM = diabetes mellitus. ASCVD = atherosclerotic cardiovascular disease. Some studies limited analyses to a safety population. Treatment effects for lower limb amputation estimated from numbers of events for several trials (DAPA−HF, EMPEROR−REDUCED, SOLOIST−WHF, EMPEROR−PRESERVED, VERTIS CV, DAPA−CKD and SCORED). Where unreported, event rates for lower limb amputation are estimated from median follow−up and other reported information (DAPA−HF, EMPEROR−REDUCED, SOLOIST−WHF, EMPEROR−PRESERVED, DECLARE−TIMI58, VERTIS CV, CREDENCE, DAPA−CKD and SCORED). Treatment effects for bone fracture estimated from numbers of events for several trials (DAPA−HF, EMPEROR−REDUCED, SOLOIST−WHF, EMPEROR−PRESERVED, VERTIS CV, DAPA−CKD and SCORED). Where unreported, event rates for bone fracture are estimated from median follow−up and other reported information (DAPA−HF, EMPA−REDUCED, SOLOIST−WHF, EMPEROR−PRESERVED, EMPA−REG OUTCOME, DECLARE−TIMI58, VERTIS CV, DAPA−CKD and SCORED)

Supplemental figure 8: Effect of SGLT2 inhibitors on URINARY TRACT INFECTION, by patient group and by trial

	Number of events/ participants	Rate per 1000 patient years SGLT-2i	Placebo	Relative risk (95% CI)
**Heart failure**				
DAPA-HF				
EMPEROR-REDUCED	174/3726	37	33	1.10 (0.82-1.47)
SOLOIST-WHF	96/1216	115	96	1.19 (0.81-1.75)
EMPEROR-PRESERVED	540/5985	45	37	1.22 (1.04-1.43)
**All heart failure trials**	**810/10927**			**1.19 (1.04-1.36)**
**Type 2 DM at high ASCVD risk**				
EMPA-REG OUTCOME	1265/7020	58	58	0.96 (0.85-1.08)
CANVAS Program	443/10142	40	37	1.10 (0.90-1.35)
DECLARE-TIMI58	260/17143	3.5	3.7	0.93 (0.73-1.18)
VERTIS CV	945/8238	40	34	1.19 (1.05-1.36)
**All type 2 DM at high ASCVD risk trials**	**2913/42543**			**1.05 (0.97-1.13)**
**Chronic kidney disease**				
CREDENCE	466/4397	48	45	1.08 (0.90-1.29)
DAPA-CKD				
SCORED	1195/10577	86	83	1.04 (0.94-1.16)
**All chronic kidney disease trials**	**1661/14974**			**1.05 (0.96-1.15)**
**OVERALL**	**5384/68444**			**1.07 (1.02-1.13)**

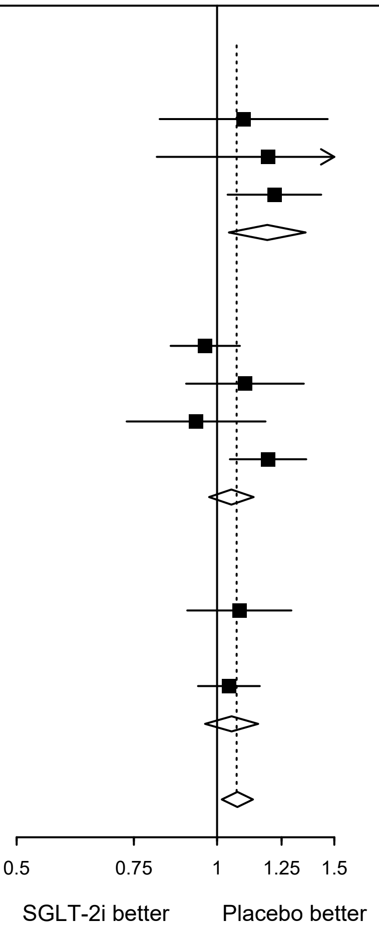

Heterogeneity between trials within patient groups:
Heart failure p=0.82;
Type 2 DM at high ASCVD risk p=0.07;
Chronic kidney disease: p=0.74

Heterogeneity between 3 patient groups: p=0.25

DM = diabetes mellitus. ASCVD = atherosclerotic cardiovascular disease. Some studies limited analyses to a safety population. DAPA-HF and DAPA -CKD not included as numbers of events (or aggregated numbers across multiple Preferred Terms) unavailable. Urinary tract infection outcomes generally included serious and non-serious advsere events. Treatment effects estimated from numbers of events for several trials (EMPEROR-REDUCED, SOLOIST-WHF, EMPEROR-PRESERVED, DECLARE-TIMI58, VERTIS CV and SCORED). Where unreported, event rates are estimated from median follow-up and other reported information (EMPEROR-REDUCED, SOLOIST-WHF, EMPEROR-PRESERVED, EMPA-REG-OUTCOME, DECLARE-TIMI58, VERTIS CV and SCORED).

Supplemental figure 9: Effect of SGLT2 inhibitors on (a) MYCOTIC GENITAL INFECTION and (b) FOURNIER'S GANGRENE, by patient group and by trial

	MYCOTIC GENITAL INFECTION				FOURNIER'S GANGRENE			
	Number of events/ participants	Rate per 1000 patient years SGLT2i	Rate per 1000 patient years Placebo	Relative risk (95% CI)	Number of events/ participants	Rate per 1000 patient years SGLT2i	Rate per 1000 patient years Placebo	Relative risk (95% CI)
**Heart failure**								
DAPA−HF					1/4736	0	<0.1	0.14 (0.00−6.82)
EMPEROR−REDUCED	43/3726	12	4.8	2.58 (1.33−5.01)	1/3730	<0.1	0	7.40 (0.15−373.21)
SOLOIST−WHF	6/1216	11	2.2	5.05 (0.59−43.10)				
EMPEROR−PRESERVED	89/5985	10	3.4	3.04 (1.88−4.91)	16/5985	1.2	1.2	1.00 (0.37−2.66)
**All heart failure trials**	**138/10927**			**2.93 (2.00−4.29)**	**18/14451**			**1.00 (0.40−2.52)**
**Type 2 DM at high ASCVD risk**								
EMPA−REG OUTCOME	343/7020	21	5.8	3.55 (2.57−4.91)	0/7020	0	0	
CANVAS Program	699/10142	45	13	3.90 (3.12−4.87)				
DECLARE−TIMI58	85/17143	2.1	0.3	8.36 (4.19−16.68)	6/17160	0	0.1	0.26 (0.05−1.31)
VERTIS CV	339/8238	18	5.1	3.53 (2.57−4.87)	0/8246	0	0	
**All type 2 DM at high ASCVD risk trials**	**1466/42543**			**3.88 (3.32−4.53)**	**6/17160**			**0.26 (0.05−1.31)**
**Chronic kidney disease**								
CREDENCE	63/4397	9.7	2.5	3.20 (1.70−6.02)				
DAPA−CKD					1/4298	0.2	0	7.39 (0.15−372.41)
SCORED	170/10577	18	6.4	2.78 (1.98−3.89)				
**All chronic kidney disease trials**	**233/14974**			**2.86 (2.12−3.86)**	**1/4298**			**7.39 (0.15−372.41)**
**OVERALL**	**1837/68444**			**3.54 (3.11−4.03)**	**25/35909**			**0.79 (0.36−1.72)**

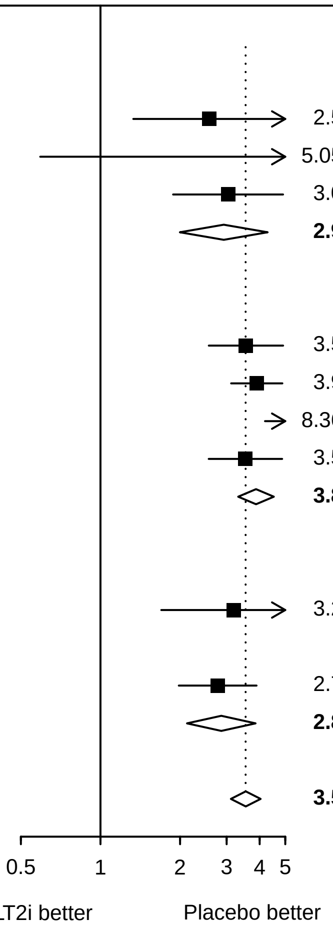

Heterogeneity between trials within patient groups:
Heart failure p=0.82;
Type 2 DM at high ASCVD risk p=0.15;
Chronic kidney disease: p=0.7

Heterogeneity between 3 patient groups: p=0.12

DM = diabetes mellitus. ASCVD = atherosclerotic cardiovascular disease. Some studies limited analyses to a safety population. Where sex−specific treatment effects and event rates were reported for mycotic genital infection, an inverse−variance weighted average was calculated (CANVAS Program and CREDENCE). Treatment effects for mycotic genital infection estimated from numbers of events for several trials (EMPEROR−REDUCED, SOLOIST−WHF, EMPEROR−PRESERVED, VERTIS CV and SCORED). Where unreported, event rates for mycotic genital infection are estimated from median follow−up and other reported information (EMPEROR−REDUCED, SOLOIST−WHF, EMPEROR−PRESERVED, EMPA−REG OUTCOME, DECLARE−TIMI58, VERTIS CV and SCORED). Formal peer−reviewed published results for Fournier's gangrene unavailable for SOLOIST−WHF, CANVAS Program, CREDENCE and SCORED. EMPEROR−PRESERVED data for Fournier's gangrene uses reports of 'complicated genital infections'. Due to rarity of Fournier's gangrene, Peto's method was used to combine results from trials. Trials with no events in either arm were not included in the meta−analysis.

Supplemental figure 10: Effect of SGLT2 inhibitors on ACUTE KIDNEY INJURY, by patient group and by trial

	Number of events/ participants	Rate per 1000 patient years SGLT-2i	Rate per 1000 patient years Placebo	Relative risk (95% CI)
**Heart failure**				
DAPA-HF	69/4744	6.4	13	0.50 (0.30-0.82)
EMPEROR-REDUCED	113/3730	19	28	0.66 (0.45-0.96)
SOLOIST-WHF	52/1216	55	59	0.94 (0.55-1.59)
EMPEROR-PRESERVED				
**All heart failure trials**	**234/9690**			**0.66 (0.51-0.86)**
**Type 2 DM at high ASCVD risk**				
EMPA-REG OUTCOME	82/7020	2.5	6.2	0.41 (0.27-0.63)
CANVAS Program	58/10142	1.6	2.5	0.66 (0.39-1.11)
DECLARE-TIMI58	300/17143	3.5	4.9	0.69 (0.55-0.87)
VERTIS CV	64/8238	2.5	2.7	0.95 (0.57-1.59)
**All type 2 DM at high ASCVD risk trials**	**504/42543**			**0.65 (0.55-0.78)**
**Chronic kidney disease**				
CREDENCE	184/4397	17	20	0.85 (0.64-1.13)
DAPA-CKD	91/4298	7.6	10	0.75 (0.50-1.13)
SCORED				
**All chronic kidney disease trials**	**275/8695**			**0.82 (0.65-1.03)**
**OVERALL**	**1013/60928**			**0.70 (0.62-0.79)**

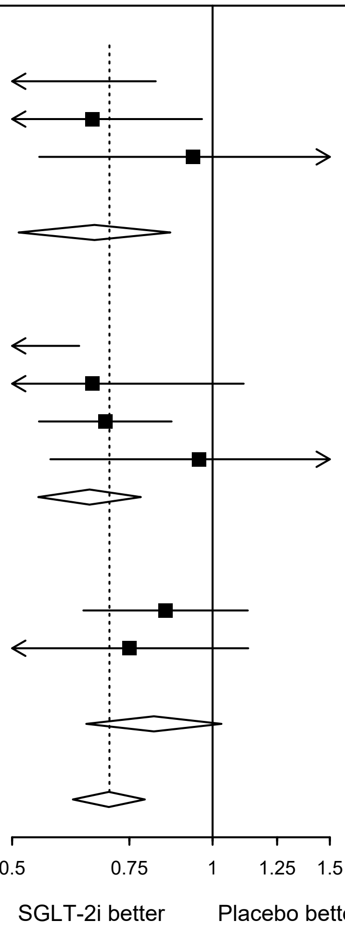

Heterogeneity between trials within patient groups:
Heart failure p=0.24;
Type 2 DM at high ASCVD risk p=0.08;
Chronic kidney disease: p=0.62

Heterogeneity between 3 patient groups: p=0.3

DM = diabetes mellitus. ASCVD = atherosclerotic cardiovascular disease. Treatment effects for acute kidney injury estimated from numbers of events for DAPA-HF, SOLOIST-WHF, EMPA-REG OUTCOME, VERTIS CV and DAPA-CKD. Where unreported, event rates for acute kidney injury are estimated from follow-up duration and other reported information for DAPA-HF, SOLOIST-WHF, EMPA-REG OUTCOME, DECLARE-TIMI58, VERTIS CV and DAPA-CKD. Some studies limited analyses for acute kidney injury to a safety population. Results for acute kidney injury from SCORED and EMPEROR-PRESERVED unavailable. EMPA-REG OUTCOME also reported effects on a standard MedDRA query of Acute Renal Failure: 246/4687 (rate 17) vs 155/2333 (rate 21), RR=0.79 (0.65-0.96). EMPEROR-PRESERVED also reported effects on Acute Renal Failure: 363/2996 (rate 56) vs 384/2989 (rate 59), RR=0.94 (0.82-1.08).
